# Optimising Cell Aggregate Expansion in a Perfused Hollow Fibre Bioreactor via Mathematical Modelling

**DOI:** 10.1371/journal.pone.0105813

**Published:** 2014-08-26

**Authors:** Lloyd A. C. Chapman, Rebecca J. Shipley, Jonathan P. Whiteley, Marianne J. Ellis, Helen M. Byrne, Sarah L. Waters

**Affiliations:** 1 Mathematical Institute, University of Oxford, Oxford, United Kingdom; 2 Department of Mechanical Engineering, UCL, London, United Kingdom; 3 Department of Computer Science, University of Oxford, Oxford, United Kingdom; 4 Department of Chemical Engineering, University of Bath, Bath, United Kingdom; University of Southampton, United Kingdom

## Abstract

The need for efficient and controlled expansion of cell populations is paramount in tissue engineering. Hollow fibre bioreactors (HFBs) have the potential to meet this need, but only with improved understanding of how operating conditions and cell seeding strategy affect cell proliferation in the bioreactor. This study is designed to assess the effects of two key operating parameters (the flow rate of culture medium into the fibre lumen and the fluid pressure imposed at the lumen outlet), together with the cell seeding distribution, on cell population growth in a single-fibre HFB. This is achieved using mathematical modelling and numerical methods to simulate the growth of cell aggregates along the outer surface of the fibre in response to the local oxygen concentration and fluid shear stress. The oxygen delivery to the cell aggregates and the fluid shear stress increase as the flow rate and pressure imposed at the lumen outlet are increased. Although the increased oxygen delivery promotes growth, the higher fluid shear stress can lead to cell death. For a given cell type and initial aggregate distribution, the operating parameters that give the most rapid overall growth can be identified from simulations. For example, when aggregates of rat cardiomyocytes that can tolerate shear stresses of up to 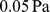 are evenly distributed along the fibre, the inlet flow rate and outlet pressure that maximise the overall growth rate are predicted to be in the ranges 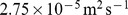 to 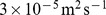 (equivalent to 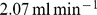 to 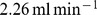) and 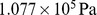 to 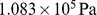 (or 15.6 psi to 15.7 psi) respectively. The combined effects of the seeding distribution and flow on the growth are also investigated and the optimal conditions for growth found to depend on the shear tolerance and oxygen demands of the cells.

## Introduction

**Figure 1:**
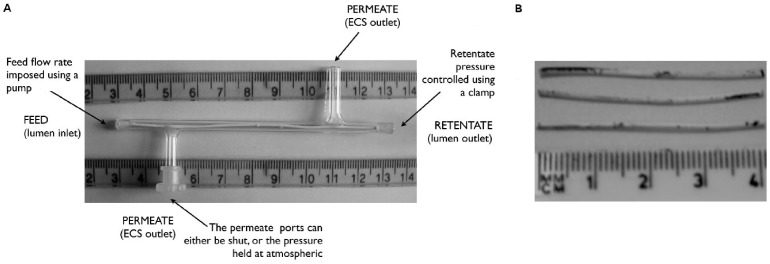
Hollow fibre bioreactor and cell distribution following culture. **A**, Photograph of single-fibre HFB module. ECS  =  extra-capillary space. Adapted from [1]. **B**, Uneven distribution of pZIP osteogenic cells on a poly(lactic-co-glycolic) acid hollow fibre 7 days after seeding. Dark patches are methylene-blue-stained cells. Top section is from the outlet end of the fibre, middle section from the middle of the fibre and bottom section from the inlet end with the flow going from right to left. Photo reproduced from [2] with permission.

*In vitro* tissue engineering that uses cells extracted from the patient to grow tissues in the laboratory offers a viable alternative to donor transplants for replacing tissue that has been damaged or lost. However, to grow substitutes of clinically relevant dimensions it is necessary first to expand the population of cells from the patient. Hollow fibre bioreactors (HFBs) are promising cell expansion devices: they consist of a glass module containing a single or multiple hollow porous biodegradable polymer fibre(s) onto (or around) which the cells are seeded. In this study, we consider the single-fibre HFB shown in Figure 1A. The outer surface of the fibre provides a large area (relative to the volume of the device) for cell proliferation. The flow rate of culture medium into the lumen, 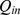, is prescribed and the relative fluid fluxes through the fibre wall (or membrane) and out of the lumen are controlled by a back pressure, 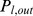, applied at the lumen outlet. This enables the nutrient and fluid shear stress environment of the cell population to be controlled to encourage functional growth. Two outlets from the extra-capillary space (ECS), the space around the fibres, can be closed or opened to promote flow through the membrane and improve nutrient delivery to the cells and removal of waste products [1].

Despite these advantages, the combined effects of the flow conditions, nutrient transport and initial cell seeding distribution on cell population expansion are not well understood. Increasing the flow through the membrane improves nutrient delivery to the cells but also increases the fluid shear stress they experience. Whilst higher nutrient concentration promotes cell proliferation and higher shear stress can stimulate proliferation, excess shear stress can lead to cell death. Hence it is necessary to find the flow parameters and seeding distribution that give the optimal balance of nutrient delivery and shear stress for the given cell type.

Cells are typically seeded onto the surface of the fibre by injecting an inoculum via the ECS outlets and fixing the module to a drum rotating at 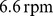 for 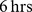 [2]. This reduces cell damage and enhances cell adhesion compared to pumping the inoculum through the ECS. However, the cell distribution over the surface after culturing is often uneven (Figure 1B) and the yield poor compared with that achieved on flat tissue culture plastic [2]. During seeding, cells attach to the membrane individually or in small groups, but many cell populations grow predominantly as aggregates [2]–[5]. Experimental and modelling studies have sought to explain the interactions between the flow of culture medium, initial cell distribution and cell population growth in specific perfusion bioreactor systems (see [6] for a review). Although general relationships have been identified, they typically pertain to cells seeded in 3D scaffolds, so have limited applicability to the HFB. Indeed, no study has considered the effect of cell seeding on cell proliferation in a HFB. While it is possible to measure the nutrient concentration globally in HFB experiments, for example at the inlet and outlets, there is a lack of detailed spatial information on local nutrient levels, fluid shear stress and cell distribution inside the bioreactor.

These issues motivate the development of the following mathematical model, in which we consider aggregates of cells that are a single cell thick growing along the outer surface of the fibre in a HFB. The growth rate of the aggregates depends on the local oxygen concentration (as oxygen is considered to be the growth-rate-limiting nutrient [1]) and the shear stress exerted on them by the flow (following [7], [8]). We determine optimal operating conditions (lumen inlet flow rate and outlet pressure) for growth of the aggregates to confluence for different cell types, and investigate the impact of the seeding distribution on the aggregate growth rate.

Numerous experimental and theoretical studies of fluid and nutrient transport in HFBs have been carried out (see [9]). Theoretical studies have typically focussed on fluid and mass transport in a single-fibre unit (referred to as a Krogh cylinder). In the lumen these are described by the Stokes and advection-diffusion equations respectively, while in the membrane and ECS convective effects are typically ignored and mass transport is assumed to be diffusion-dominated. This is representative of the setup with the ECS outlets closed. If present, cells are usually taken to be seeded in gel throughout the ECS so that nutrient uptake can be modelled by a reaction term in the ECS mass transport equation. This reaction term has followed zeroth-order, first-order or full nonlinear Michaelis-Menten kinetics depending on the nutrient demands of the cells, and the nutrient transport equations have been solved via appropriate analytical or numerical methods (see [9] for a review). Shipley and Waters [1] adopted an analytical approach to model fluid and nutrient transport in a single-fibre HFB, with cells seeded in gel in the ECS. Their work represents a departure from previous studies by considering nutrient advection in the membrane and ECS (representative of open ECS outlets and/or an applied downstream lumen pressure), using Darcy's Law and reaction-advection-diffusion equations to describe the fluid flow and mass transport respectively. Growth of the cell population and its effect on the permeability of the ECS to fluid and nutrient were neglected. This is typical of theoretical studies of HFBs, which focus on timescales associated with transport processes rather than those associated with cell proliferation, and model the ECS as devoid of cells or packed with cells already at physiological densities, so that proliferation may be neglected [10]. The models of Brotherton and Chau [10] and Mohebbi-Kalhori et al. [11], in which Monod kinetics were used to describe nutrient-dependent cell proliferation in the ECS, are notable exceptions. Brotherton and Chau also included feedback of the cell proliferation on the fluid flow and nutrient uptake by making the ECS fluid permeability and nutrient uptake functions of the cell density.

None of these studies, however, have considered the influence of fluid shear stress on cell proliferation. Experimental studies have investigated the effect of fluid shear stress on the differentiation and proliferation of bone cell progenitors (see [12], [13] for comprehensive reviews), which are commonly cultured in HFBs. Computational approaches have been used to assess the role of shear stress in tissue construct development [8], [14]–[16]. Shakeel et al. [8] modelled shear-stress-dependent cell proliferation and nutrient transport in a perfusion bioreactor. They adapted a functional form proposed by O′Dea et al. [7] to describe elevated proliferation at intermediate shear stress levels, and used a similar function to describe faster nutrient uptake at intermediate shear stresses. Korin et al. [15] combined modelling and experiments to investigate the effect of oxygen transport and shear stress on human foreskin fibroblast proliferation in a microchannel bioreactor. Cell proliferation was governed by a logistic growth law and oxygen uptake at the cell surface described via Michaelis-Menten kinetics, which were independent of the changing cell density.

The model presented here differs from previous studies of HFBs in a number of key respects. We assume that cells are attached to the outer surface of the fibre rather than seeded throughout the ECS, so the model has greater relevance to cell population expansion. Cell proliferation and death are modelled phenomenologically by nutrient- and shear-stress-dependent elongation and shortening of the cell aggregates. This enables us to determine the impact of changes in the operating parameters and seeding conditions on the growth rate of the aggregates, rather than just on the fluid and mass transport through the bioreactor. Additionally, we account for feedback on the flow and nutrient distribution from cell proliferation and death (via changes in the permeability of the outer membrane surface and oxygen uptake with aggregate growth).

The structure of the paper is as follows. In the Methods section, we present the governing equations for fluid and oxygen transport and cell aggregate growth, and identify values for the transport parameters. We briefly describe how the model is parameterised for specific cell types, simplified and solved numerically (further information is given in File S1). In the Results section we perform a parameter sensitivity analysis, assessing the impact of changing the inlet flow rate, outlet pressure and initial aggregate distribution on the aggregate growth, and identify optimal operating conditions that minimise time to confluence for a specific cell type. We summarise our findings and explain how they may be used to maximise the cell yield for different cell types in the Discussion section.

## Methods

### Model Setup

**Figure 2:**
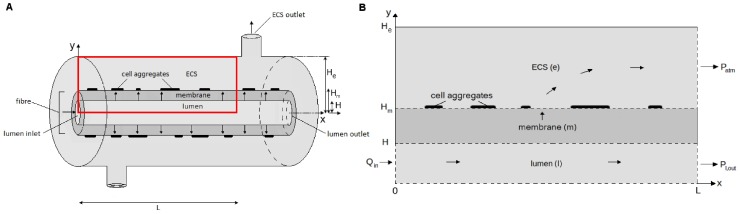
Schematics of hollow fibre bioreactor and model setup. **A**, Schematic of the laboratory single-fibre HFB module. **B**, Schematic of model setup. Cell aggregates on membrane shown in black. Arrows show direction of fluid flow. Red rectangle in **A** shows the region that **B** corresponds to. 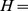 lumen radius, 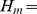 membrane radius, 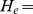 ECS radius, 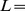 length of model domain, 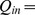 lumen inlet flow rate, 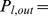 lumen outlet pressure, and 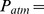 atmospheric pressure.

A schematic of the HFB is shown in Figure 2A. We consider a representative 2D cross-section as shown in Figure 2B. We assume that the ECS outlet nearest the lumen inlet is closed while that nearest the lumen outlet is open. Culture medium is pumped into the lumen at a prescribed rate, 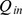, and flows out of the bioreactor via the lumen or the open ECS outlet. The pressure imposed at the lumen outlet, 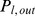, determines the ratio of flow through the lumen to that through the membrane into the ECS. All external boundaries other than the inlet and outlets are impermeable to fluid and oxygen. The oxygen concentration in the culture medium pumped into the inlet, 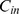, is constant. Oxygen is transported through the bioreactor by advection and diffusion. For simplicity, we treat the flow and oxygen distribution as being symmetric about the lumen centreline. Cell aggregates are distributed along the outer surface of the membrane, and absorb oxygen and grow or shrink at a rate that depends on the local oxygen concentration and fluid shear stress. This change in aggregate length affects the flow and oxygen profiles along the membrane through increased/decreased oxygen uptake and reduced/increased permeability of the membrane outer surface to fluid.

### Governing Equations

#### Fluid Transport

We let 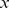 denote the axial distance down the lumen centreline, with the inlet at 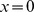 and the outlet at 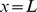, and 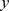 the height above the lumen centreline. The corresponding unit vectors in the 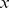
- and 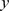-directions are denoted by 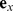 and 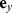. In the lumen and ECS, denoted by subscripts 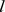 and 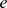 respectively, we treat the flow as quasi-steady on the timescale of aggregate growth, which is much longer than the timescale for fluid transport (days or weeks compared to seconds or minutes). We neglect inertial forces in the fluid since they are much smaller than viscous forces for experimentally-relevant parameter values (this is verified a posteriori—see the Reduced Model section). Hence, we describe the fluid flow by the steady incompressible Stokes flow equations 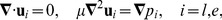
(1)where 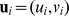, 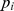 and 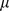 are the fluid velocity, pressure and dynamic viscosity respectively. In the porous membrane, denoted by subscript 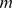, fluid transport is modelled using the incompressible Darcy flow equations 
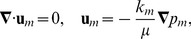
(2)where 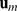 and 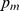 are the fluid velocity and pressure (averaged over the pore space), and 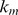 is the membrane permeability.

At the interface between the lumen and the membrane, 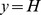, we impose continuity of normal velocity and normal stress. We make the modelling assumption that all of the lumen fluid stress is taken up by the fluid in the membrane as in previous studies [1], [17] (other constitutive relations could be considered, see [18] for a full discussion). Hence the continuity conditions at the interface are 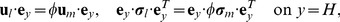
(3)where 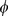 is the porosity of the membrane (assumed constant), and 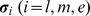 are the fluid stress tensors given by 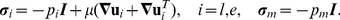
(4)


The cell aggregates on the outer surface of the membrane are taken to be infinitesimally thin (since a typical cell diameter (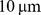) is small compared with the depth of the membrane (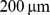) and the aggregates are one-cell thick). At the outer surface of the membrane, 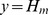, we impose continuity of normal fluid velocity but assume there is a jump in the normal stress proportional to the fluid flux, so that 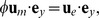


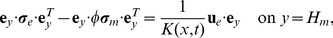
(5)where 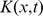 represents the permeability of the outer surface of the membrane. We assume that the aggregates provide resistance to the flow such that 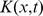 has a low value in regions covered by cell aggregates. In regions not covered by aggregates, we assume that 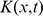 has a higher but finite value, 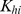, to model a reduction in surface permeability caused by extra-cellular matrix and protein deposition by the cells. The discontinuity in 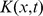 at the ends of the aggregates is smoothed in our numerical simulations as described in Section C.2 of File S1. We suppose that the cell density within an aggregate remains constant as the aggregate grows/shrinks, so that 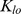 and 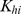 are fixed. The normal stress boundary condition in (5) corresponds to Starling's equation for the fluid flux across a thin membrane, i.e. the fluid flux being proportional to the pressure difference across the membrane (see Section A.1 in File S1).

At the interfaces between the membrane and the free fluid we impose no-slip conditions 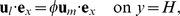


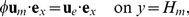
(6)since Shipley et al. [19] fitted a mathematical model that incorporated slip to experimental measurements of fluid distribution and found that slip was negligible for these membranes. We assume the flow is symmetric about the lumen centreline, so 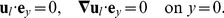
(7)


The outer boundary of the ECS is impermeable, so we impose no-slip and no-flux conditions there 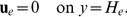
(8)


Having specified the transverse boundary conditions for the fluid flow we now specify the appropriate axial boundary conditions. There is no fluid flux out of the ends of the membrane or at the upstream end of the ECS 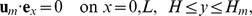


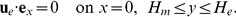
(9)


The flow rate at the inlet and the normal stress at the lumen outlet are prescribed so that 
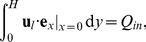
(10)

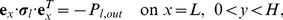
(11)where the values of 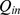 and 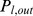 are taken from experiments. Although solving full Stokes flow would require two pointwise boundary conditions at the inlet, we only require (10) since we exploit the small aspect ratio of the fibre to simplify the flow equations (see the Reduced Model section). At the ECS outlet we impose continuity of normal stress 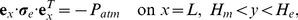
(12)where 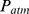 is atmospheric pressure (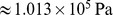 or 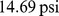).

#### Oxygen Transport

We assume that oxygen transport is quasi-steady on the timescale of aggregate growth, and governed by a steady advection-diffusion equation in each region 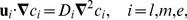
(13)where 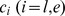 and 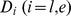 are the oxygen concentration (per unit volume of fluid) and diffusivities in the relevant domains, and 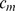 and 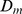 are the average oxygen concentration and diffusivity in the membrane. We assume that the diffusivities are constant.

At the lumen-membrane interface, we impose continuity of the oxygen concentration and flux 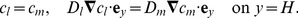
(14)


At the interface between the membrane and the ECS, we again impose continuity of the concentration, but also impose a jump in the flux across the aggregates due to cellular uptake of oxygen 
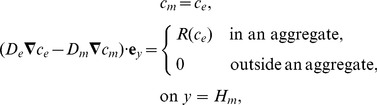
(15)where the oxygen uptake flux 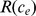 is an increasing saturating function of 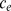


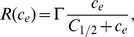
with the positive constant 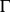 denoting the maximal oxygen uptake flux and 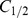 the concentration at which the flux is half-maximal.

Since the oxygen distribution is taken to be symmetric about the lumen centreline, we impose no diffusive flux through the lumen centreline 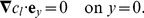
(16)


The upstream end and outer wall of the ECS and the ends of the membrane are impermeable to nutrients, so that 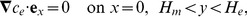
(17)

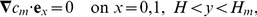
(18)

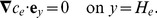
(19)


The inlet oxygen concentration is fixed, so 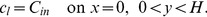
(20)


Following Shipley et al. [1], we assume that the culture medium leaving the lumen and ECS outlets is well-mixed and impose zero-diffusive-flux boundary conditions at the outlets, 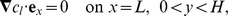


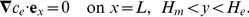
(21)


#### Cell Aggregate Growth

Net increases/decreases in the cell population are modelled by elongation/shortening of the aggregates, whose initial distribution and lengths along the membrane are prescribed. It is assumed that cell proliferation occurs over the whole length of the aggregate, and that when cells in the middle of an aggregate divide the neighboring cells move outwards to accomodate the new cells, maintaining the cell density as the aggregate grows. This is based on evidence from cell migration and proliferation assays on 2D substrates that cells distant from a growing edge continue to proliferate and exert a mitotic pressure on their neighbouring cells [20]–[23]. When two aggregates come into contact, they are assumed to coalesce and are thereafter treated as one larger aggregate. If one end of an aggregate reaches either end of the membrane (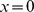 or 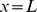), any further growth is taken to occur at its opposite end. It is assumed that when cells die they detach from the membrane and are removed with the flow, causing the aggregates to shrink. For simplicity, we assume that the effects of the oxygen concentration and fluid shear stress on the cell proliferation rate 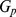 and death rate 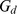, and hence on the net rate of aggregate elongation, are multiplicative (see Equation (22)). This means that the aggregates cannot grow in either hypoxic or excess shear conditions, which would be possible if the effects were assumed to be additive (e.g. if the shear stress was high enough to kill cells, but the oxygen concentration was also very high and promoted proliferation sufficiently for the net effect to be positive). Nevertheless, the modular nature of the model means that alternative growth laws (based on different assumptions, such as assuming proliferation only occurs near the aggregate ends) could easily be incorporated.

**Figure 3:**
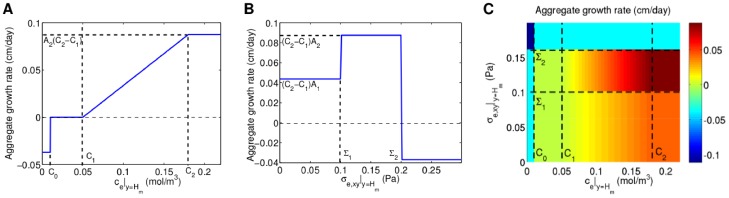
Prescribed variation in aggregate growth rate with oxygen concentration and shear stress. **A**, Variation with oxygen concentration, 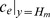, for fixed shear stress, 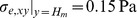. **B**, Variation with shear stress, 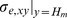, for fixed oxygen concentration, 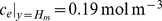. **C**, Variation with both oxygen concentration and shear stress. Concentration thresholds: 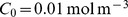, 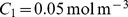 and 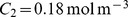. Shear stress thresholds: 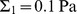, 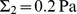. Growth rate parameters: 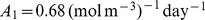, 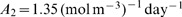, 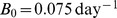, 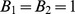.

Several authors [8], [10], [24], [25] have used Monod kinetics to describe the relationship between oxygen concentration and cell proliferation, so that the proliferation rate is approximately constant at low concentrations, increases with concentration for intermediate concentrations, and plateaus to a maximum at high concentrations. Following McElwain and Ponzo [26], we approximate Monod kinetics in a piecewise linear fashion as shown in Figure 3A, and assume that cells die below a certain oxygen concentration. Following O′Dea et al. [7], we take the cell proliferation/death rate to be a stepped function of the fluid shear stress on the cells along the membrane outer surface, 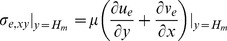 (see Section A.3 in File S1 for details on how this is approximated in the reduced model), with faster cell proliferation at intermediate shear stresses and cell death at high shear stresses as shown in Figure 3B. This relationship is based on experimental observations (see Table S6 and references therein).

If there are 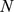 cell aggregates at time 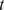, such that the 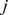
th aggregate occupies the interval 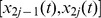 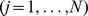, then the evolution equation for the length of the 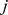
th aggregate is 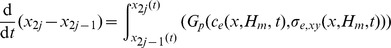


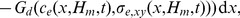
(22)where 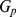 and 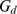 are the cell proliferation rate and death rate respectively at each point in the aggregate due to the combined effects of the oxygen concentration and shear stress (see Figure 3C). The cell proliferation and death functions are integrated over the length of the aggregate (from 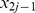 to 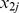) to give the net rate of change of its length.

There is little quantitative data available from which to pose and parameterise a growth law, so we use the following constitutive relationships for the proliferation and death rates to capture the key known features of how oxygen and shear stress impact growth 
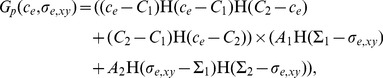
(22a)

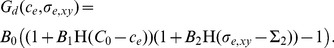
(22b)


In (22a) and (22b), 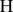 is the Heaviside function; 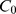 the oxygen concentration below which cells die; 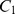 the minimum concentration for cell proliferation above which the aggregate growth rate increases linearly with 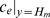; 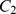 the concentration above which the aggregate growth rate is a constant maximum; 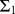 the shear stress at which the transition in the aggregate growth rate from the constant baseline rate 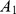 to the elevated rate 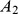 occurs; 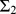 the shear stress above which cells die; and 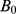, 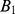, 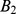 constants that together determine the constant death (aggregate shortening) rates for low concentration (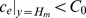), high shear stress (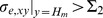) and a combination of the two (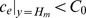, 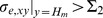). These forms of 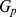 and 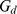 capture the fact that most cells die if they receive insufficient oxygen, remain quiescent in low oxygen conditions, proliferate at an increasing rate with increasing oxygen concentration and die if the fluid shear stress they experience is too high. Despite the lack of experimental data for parameterisation, the model can be used to test the impact of promoting either sensitivity to oxygen or shear stress, through the concentration and shear stress thresholds and growth and death rate constants.

We make the further assumption that cell division forces cells outwards in such a way that the growth at the ends of the aggregate is equal, i.e. 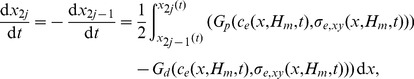
(23)except when one end of the aggregate reaches the end of the membrane, in which case there is only growth at the opposite end and the total growth is halved. This gives 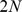 equations for the 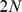 unknown positions of the aggregate ends 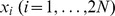. To close equations (1)–(23) we prescribe the initial distribution of aggregates via 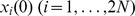.

### Reduced Model

Typical values of the model parameters, where possible taken from the literature or determined by testing in the laboratory, are given in Table 1. Equations (1)–(23) are nondimensionalised and the small aspect ratio of the fibre lumen (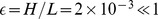), membrane and ECS exploited to simplify the system (see Section A in File S1 for details). The bounds of applicability of the reduced model are set by the values of the key dimensionless parameters—the reduced Reynolds number, reduced Péclet numbers and second Damköhler number. The reduced Reynolds number measures the ratio of inertial to viscous forces in the fluid accounting for the small aspect ratio of the lumen, and is given by 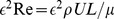, where 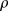 is the fluid density and 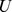 is the typical lumen fluid velocity set by the inlet flow rate 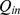. For typical inlet flow rates used in experiments, 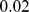
–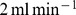 [1], [19], the reduced Reynolds number 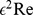 lies in the range 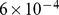
–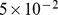 (with 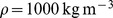, the density of water at 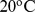 to 2 s.f. [27]), which justifies our neglect of inertia in the fluid flow equations. The reduced Péclet numbers in the different regions, 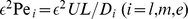, represent the ratio of the rate of axial advection of oxygen (in the 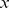-direction) to radial diffusion (in the 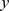-direction). For the range of flow rates given above, the reduced Péclet numbers are between 0.2 and 177 (see Table S1), so we retain terms at order 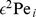 in the leading order oxygen transport equations. Finally, the second Damköhler number, 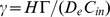, gives the ratio of the rate of oxygen uptake by the cells to the rate of diffusive transport. The jump in oxygen flux across the aggregates is assumed to balance the oxygen uptake by the cells so 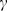 must be of order 1 for the reduced model to be valid.

**Table 1:** Fluid and oxygen transport and aggregate growth parameter values.

Parameter	Description	Typical Value(s)	Reference
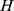	lumen radius	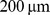	[19]
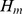	fibre outer radius	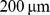	[19]
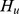	ECS outer radius	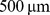	[19]
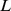	fibre length	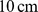	[19]
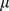	dynamic viscosity of fluid	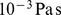	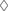
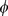	membrane porosity	0.77	[31]
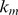	membrane permeability	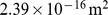	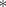
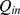	2D lumen inlet flow rate	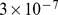 − 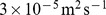	[19]
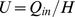	typical flow velocity	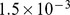 − 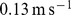	-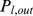	lumen outlet pressure	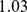 − 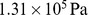	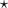 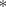
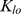	permeability of membrane outer surface + cell aggregate	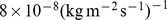	-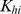	membrane outer surface permeability	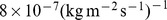	-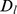	lumen oxygen diffusivity	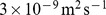	[32]
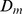	membrane oxygen diffusivity	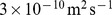	[32]
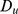	ECS oxygen diffusivity	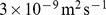	[32]
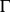	maximal oxygen uptake flux	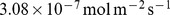	[28] 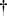
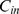	inlet oxygen concentration	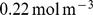	[28] 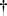
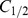	half maximal uptake rate concentration	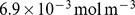	[28] 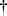
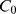	minimum oxygen concentration for cell survival	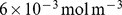	[28] 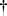
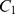	minimum oxygen concentration for cell proliferation	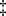	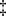
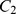	maximal growth rate oxygen concentration	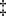	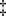
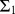	shear stress threshold for elevated proliferation rate	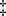	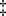
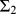	shear stress threshold for cell death	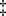	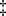
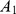	baseline growth rate for 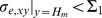	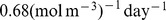	[2]
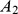	growth rate for 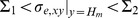	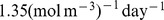	-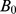	baseline aggregate shortening rate for 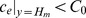 or 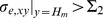	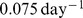	-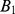	weight factor for aggregate shortening rate for 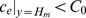	1	-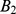	weight factor for aggregate shortening rate for 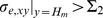	1	-

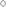
 Value for water at 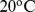 (from http://physics.info/viscosity/).

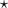
 In all simulations 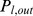 is chosen to ensure that there is no backflow at the lumen outlet.

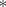
 Determined in experiments performed by L.A.C. Chapman, R.J. Shipley and M.J. Ellis at the Chemical Engineering Department, University of Bath.

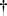
 Values stated are for rat cardiomyocytes (see Tables S2 and S4 for values for other cell types).

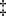
 Parameter values for which there is limited experimental evidence (see Table S6 for data on shear stress thresholds) and that are varied in the aggregate growth simulations.

Many other model parameters vary with cell type: the inlet oxygen concentration, 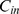; the half maximal uptake flux concentration, 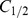; the concentration and shear stress thresholds for the aggregate growth, 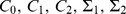; and the growth rate parameters, 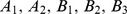. Experimental data for maximal oxygen uptake rates and 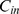, 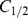 and 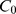 values for different cell types is presented in Tables S2 and S4 respectively. In the aggregate growth simulations in the Results section we follow Shipley et al. [9] and use values of the transport and uptake parameters appropriate to rat cardiomyocytes from the study of Radisic et al. [28] (see Table 1). We convert Radisic et al.'s estimate of the maximal volumetric oxygen uptake rate of the rat cardiomyocytes to a value for the maximal oxygen uptake flux, 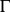, as follows 
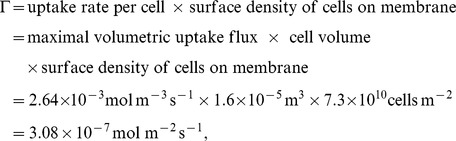
where the surface density of cells on the membrane is taken from [29] and the average cell volume estimated from the data in Table S3 (see Table S2 for further details).

Since experimental data for the concentration and shear stress thresholds is sparse (see Tables S5 and S6 for data for different shear stress regimes), a range of values is used in the simulations to determine their effect on the aggregate growth. By comparing predictions for aggregate growth from these simulations with experimental results, the relative oxygen concentration and shear stress sensitivity of the cells can be inferred. Alternatively, experiments can be performed to measure the thresholds for a particular cell type and the model used to predict how the aggregates will grow. The baseline aggregate growth rate, 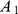, is estimated from measurements of population doubling times of human bone marrow stromal cell culture in a HFB [2] (see Section B.2.2 in File S1 for details).

### Numerical Methods

The reduced system of equations for the flow, oxygen transport and cell aggregate growth is solved using code developed in the numerical programming software MATLAB (distributed by Mathworks Inc., see http://www.mathworks.co.uk for details). A description of the algorithm is given in Section C.3 of File S1. The code was validated by comparing numerical solutions of the reduced model without aggregate growth to solutions of the full fluid and oxygen transport model (given by equations (1)–(16)) computed using the finite element software COMSOL Multiphysics 4.3 (distributed by COMSOL Inc., see http://www.comsol.com for details). Over the range of parameter values for which the approximation in the reduced model is valid, the error in the reduced model solutions was consistent with the size of terms neglected (results not shown).

## Results

### Example Oxygen, Shear Stress and Aggregate Distributions

**Figure 4:**
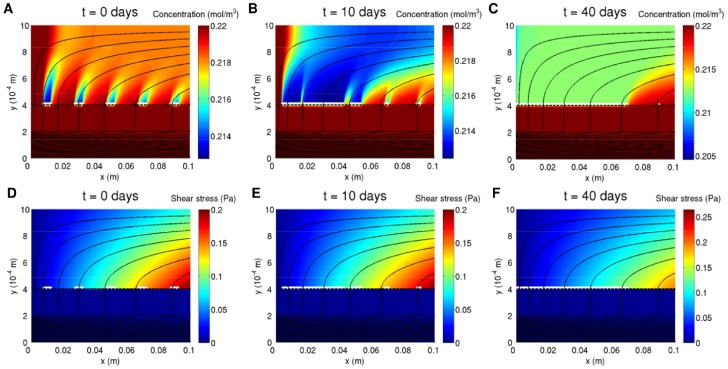
Aggregate distribution with oxygen and shear stress distributions at different time points in a growth simulation. **A-C**, Aggregate distribution with oxygen distribution. **D-F**, Aggregate distribution with shear stress distribution. Aggregates shown in white. Solid black lines are flow streamlines. The dark blue in the lumen and membrane in **D–F** indicates very low and negative fluid shear stress in these regions respectively. Parameter values: 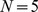, 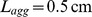, 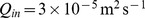, 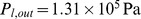, 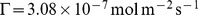, 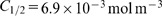, 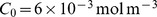, 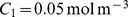, 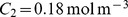, 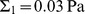, 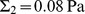, 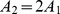, 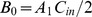, 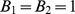. All other parameter values as in Table 1.

Figure 4 shows the distribution of rat cardiomyocyte aggregates along the membrane with the corresponding oxygen and shear stress distributions in the ECS at different time points in a growth simulation for candidate parameter values (see figure legend). The initial aggregate distribution (shown in Figures 4A,D) consists of five aggregates of length 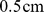 distributed evenly along the membrane. It is used for all subsequent simulations, unless otherwise stated, so that the impact of oxygen versus fluid shear stress on growth can be isolated and compared. Due to uptake by the cells, oxygen is depleted in regions that surround each aggregate and extend outwards from the membrane into the ECS. These regions increase in size as the aggregates grow and eventually merge to form one large region (Figures 4A–C). The flow of the culture medium ensures that the oxygen concentration remains highest at the upstream end of the ECS until the aggregates come into contact with it. The shear stress profile along the membrane remains fairly constant in time as the aggregates grow (Figures 4D–F), because the aggregates have only a small effect on the membrane outer surface permeability (for the chosen values of 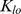 and 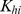). The aggregates do not reach confluence after 40 days (the longest culture period reported in the literature [30]) as the shear stress exceeds the cell death threshold (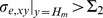) towards the outlet. Thus the three aggregates nearest the inlet grow and coalesce to form one large aggregate, while the fourth aggregate remains static and the rightmost one shrinks.

### Influence of Flow on Aggregate Growth

#### Impact of Flow on Oxygen Flux and Shear Stress

To understand how the inlet flow rate, 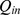, and outlet pressure, 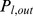, affect the growth of the aggregates it is necessary to determine how they affect the oxygen concentration around, and shear stress on, the aggregates.

**Figure 5:**
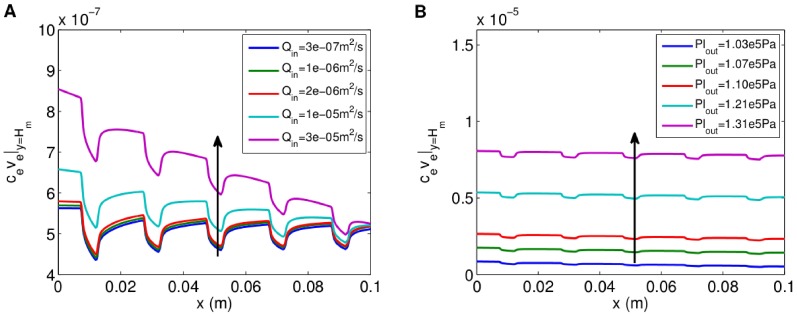
Advective oxygen flux across membrane outer surface for different flow rates and outlet pressures for rat cardiomyocyte aggregates. **A**, Advective flux, 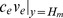, for different flow rates. Arrow shows direction of increasing 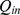 (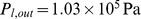). **B**, Flux for different outlet pressures. Arrow shows direction of increasing 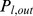 (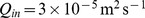). Initial aggregate distribution as in Figure 4A. Parameter values: 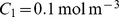, 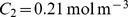, 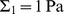, 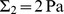, 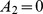, 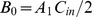, 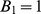, 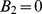. All other parameter values as in Figure 4.

**Figure 6:**
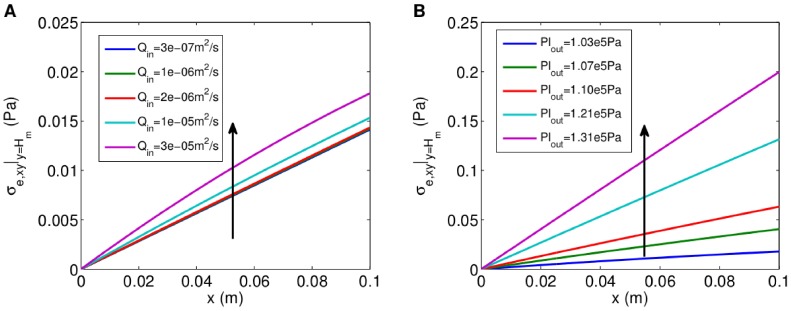
Shear stress along membrane for different flow rates and outlet presures. **A**, Shear stress for different flow rates. Arrow shows direction of increasing 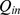 (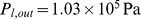). **B**, Shear stress for different outlet pressures. Arrow shows direction of increasing 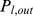 (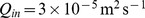). All other parameter values as in Figure 5.

Figures 5A and 5B show how the advective oxygen flux through the outer surface of the membrane, 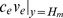, changes when 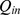 and 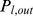 are varied for the static rat cardiomyocyte aggregate distribution. The flux dips in the regions of the membrane covered by aggregates because of the oxygen uptake and reduced fluid permeability there. Increasing 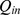 or 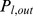 increases the oxygen flux through the membrane. However, the system is more sensitive to changes in 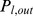 than 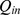: a 100-fold increase in 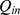 (from 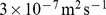 to 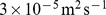) leads to an increase of at most 50% in the flux, while a 14-fold increase in 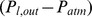 (the outlet pressure relative to atmospheric pressure, from 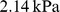 to 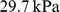, or 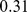 to 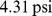) produces an 8-fold increase. This is because the bulk of the pressure drop from the lumen inlet to the ECS outlet occurs across the membrane, so increasing 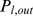 gives a much bigger pressure gradient across, and therefore flow through, the membrane. Similarly, the shear stress, 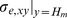, is found to increase with distance along the membrane (from 0 at the upstream end, which is closed) and as 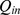 and 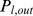 are increased, the effect being more pronounced for changes in 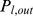 than 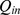 (see Figure 6). The 100-fold increase in 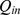 produces a 26% increase in the maximum shear stress over the membrane, whereas the 14-fold increase in 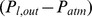 causes a 10-fold increase in the maximum shear stress.

#### Impact of Flow on Aggregate Growth

First we distinguish the effects of oxygen and shear stress on aggregate growth as 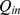 and 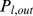 are varied by comparing simulations for which growth is purely oxygen-dependent with those for which it is purely shear-stress-dependent. Oxygen-dependent growth is appropriate to cell types such as endothelial cells and hepatocytes, which are tolerant to high shear stresses or have high oxygen demands, while shear-stress-dependent growth is appropriate to cell types such as chondrocytes and mouse embryonic stem cells, which have low oxygen demands or are sensitive to changes in shear stress (see Tables S2 and S6). In the model, purely oxygen-dependent growth is achieved by setting the shear stress growth parameters 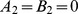 in Equation (23) and choosing 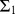 such that 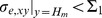 always; and purely shear-stress-dependent growth is achieved by taking 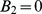 in Equation (23) and 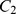 low enough such that 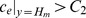 always. Following this, we consider aggregate growth for cells that are sensitive to both oxygen levels and shear stress, choosing the concentration thresholds 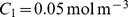 and 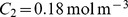 in Equation (23) such that the growth rate is linearly proportional to the concentration over a wide range and using low values for the shear stress thresholds 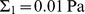 and 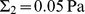. Oxygen- and shear-stress-dependent growth is relevant to cell types such as cardiomyocytes, mesenchymal stem cells and fibroblasts (see Tables S2 and S6).

Simulations are run until either the aggregates reach confluence or 40 days have elapsed. In the former case the confluence time, 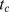, is recorded, in the latter case the total length of the aggregates, 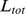, is recorded (at confluence 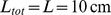).

#### Oxygen-dependent Growth

**Figure 7:**
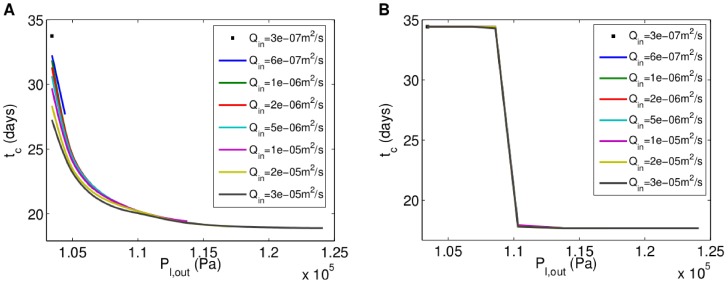
Relationship between confluence time and outlet pressure for different flow rates for rat cardiomyocyte aggregates. **A**, Relationship for oxygen-concentration-dependent growth. **B**, Relationship for shear-stress-dependent growth. Time step: 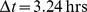 (dimensionless time step 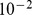). Parameter values in **A**: 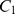, 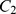, 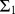, 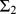, 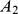, 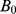, 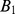, 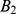 all as in Figure 5. Parameter values in **B**: 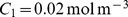, 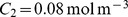, 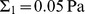, 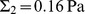, 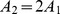, 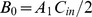, 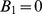, 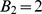. All other parameter values as in Figure 4. Curves stop as backflow occurs at higher outlet pressures.

Figure 7A shows how the confluence time, 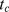, varies with 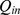 and 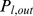 for simulated rat cardiomyocyte aggregate growth. As 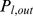 increases with 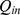 fixed, the curves end at the maximum outlet pressure for which there is no backflow. As expected, 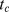 decreases as 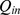 and 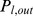 increase, the effect being more marked for changes in 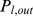. This is because increasing 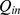 or 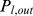 increases the advective flux of oxygen through the membrane and therefore the oxygen concentration around the aggregates and their growth rate.

#### Shear-stress-dependent Growth

The results presented in Figure 7B reveal that as 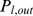 increases there is a sharp decrease in the confluence time at around 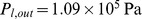 (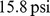), but that changes in 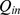 have little effect on 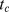. As 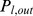 increases through 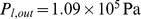, it is observed in the simulations that the number of aggregates on which the shear stress is higher than 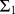 increases sharply, leading to a significant increase in the net aggregate growth. The effect is barely noticeable when 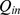 is increased, because the associated increase in the fluid flux through the membrane is much smaller.

#### Combined Oxygen-concentration- and Shear-stress-dependent Growth

**Figure 8:**
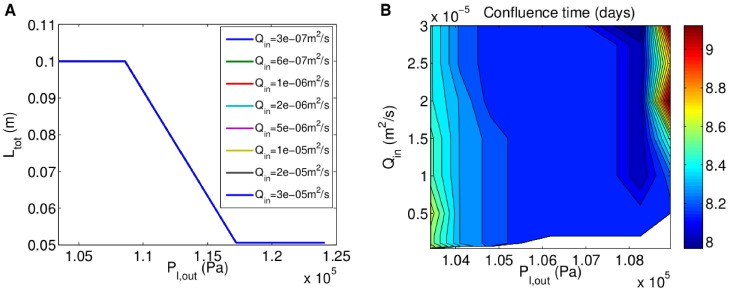
Oxygen-concentration- and shear-stress-dependent growth of shear-sensitive rat cardiomyocytes. **A**, Relationship between final total aggregate length and outlet pressure for different flow rates (graphs overlap as there is no change with flow rate). **B**, Variation in confluence time with flow rate and outlet pressure for 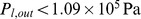. Parameter values: 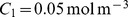, 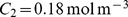, 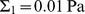, 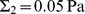, 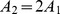, 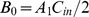, 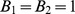. All other parameter values as in Figure 4.

Figure 8A shows that aggregates of rat cardiomyocytes with the oxygen and shear stress sensitivities given above only reach confluence when 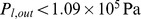, and that for 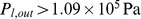, 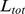 decreases as 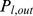 increases, up to 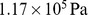 (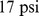) (above which it is constant). This is because at outlet pressures above 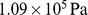 the shear stress exceeds the cell death threshold 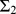 over more of the membrane and this effect outweighs the positive effect of greater oxygen delivery to the cells.

Figure 8B shows how the confluence time changes with flow rate and outlet pressure for flow rates between 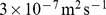 and 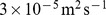 and outlet pressures ranging from 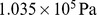 to 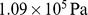 (or 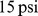 to 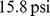) (ranges achievable with standard laboratory equipment). As 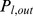 increases, the confluence time first decreases, for 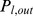 up to 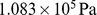 (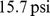), and then increases. This is because the region of the membrane in which the proliferation rate is elevated (i.e. where 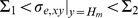) increases in extent, covering more of the aggregates, as 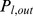 increases up to 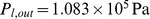, but the region in which the shear stress exceeds the death threshold 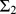 becomes larger for 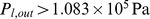. For 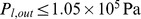 (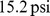), the confluence time decreases marginally as 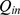 increases due to greater oxygen flux through the membrane. In the range 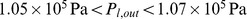 (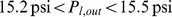), the confluence time remains approximately constant as 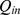 increases. For 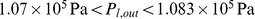, the confluence time decreases as 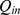 increases, reaching a minimum of 7.9 days for 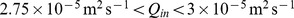 and 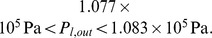 For 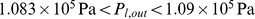 the confluence time increases with increasing 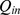, since the detrimental effects of the shear stress exceeding 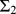 start to outweigh those of increased oxygen delivery. Hence, 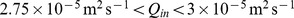 and 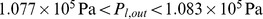 (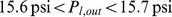) are predicted to be the optimal flow rate and outlet pressure ranges for proliferation of these shear-sensitive rat cardiomyocytes.

### Influence of Cell Seeding on Growth

**Figure 9:**
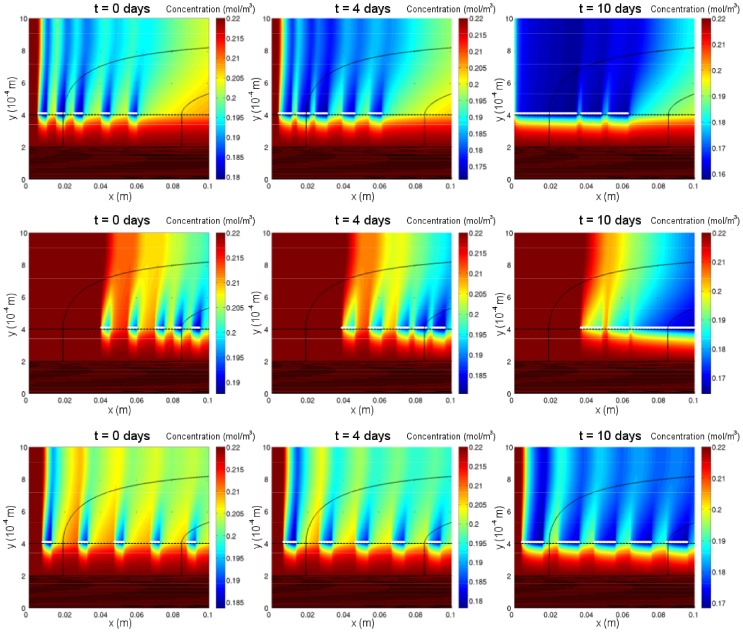
Aggregate growth and oxygen distribution at different time points for different initial aggregate distributions and low outlet pressure. First row: left-skewed distribution. Second row: right-skewed distribution. Third row: even distribution. Solid black lines are flow streamlines. Parameter values: 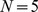, 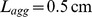, 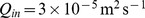, 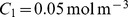, 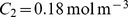, 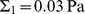, 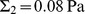, 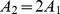, 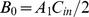, 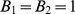. All other parameter values as in Figure 4.

Although the model can be used to simulate the growth of real seeding distributions, since seeding data is not available we use three different simple initial distributions to test the impact of skew in the distribution (towards the inlet end or outlet end) on confluence time. We expect the skew to have an effect because the oxygen and shear stress distributions that arise are spatially non-uniform and evolve as the aggregates grow. Each of the distributions has five aggregates each of initial length 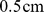, and the aggregate growth is simulated for a low outlet pressure (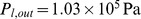) and a high outlet pressure (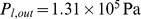). (We do not vary 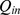 since its effect on the transmembrane fluid flux is much weaker.) The initial distributions we consider are: (i) *left-skewed* (i.e. aggregates concentrated towards the inlet); (ii) *right-skewed* (i.e. aggregates concentrated towards the outlet); and (iii) *even* (i.e. aggregates spaced uniformly across the membrane) (see 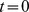 panels in Figure 9).

With the lower outlet pressure the aggregates grow to confluence in all three cases, albeit at differing rates. The aggregates reach confluence fastest if they are right-skewed (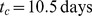), take longer if they are evenly distributed (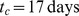), and take more than twice as long (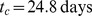) if they are left-skewed. For the right-skewed distribution the aggregates start further away from, and grow towards, the inlet. Hence, once the aggregates have merged to form one large aggregate, the end of the aggregate nearest the inlet continues to experience a high oxygen concentration as it grows, despite the weak oxygen flux through the membrane from the low outlet pressure (Figure 9). Changes in the shear stress along the membrane with the initial aggregate distribution do not have any impact on the rate of growth to confluence as the shear stress is always below the threshold for increased proliferation (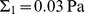) for all four distributions (results not shown).

With the higher outlet pressure, the aggregates fail to reach confluence after 40 days for all four initial distributions. The total aggregate length at 40 days is greatest for the even distribution (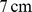) and slightly less for the left-skewed and right-skewed distributions (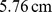 and 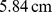 respectively). This is because when the outlet pressure is large the oxygen concentration around the aggregates is similar for the different distributions but the shear stress exceeds the threshold for cell death near the outlet end of the membrane. Aggregates at the outlet end therefore tend to shrink, so initial differences in growth between the distributions disappear over time and the aggregates merge to form one large aggregate at the inlet.

## Discussion

**Figure 10:**
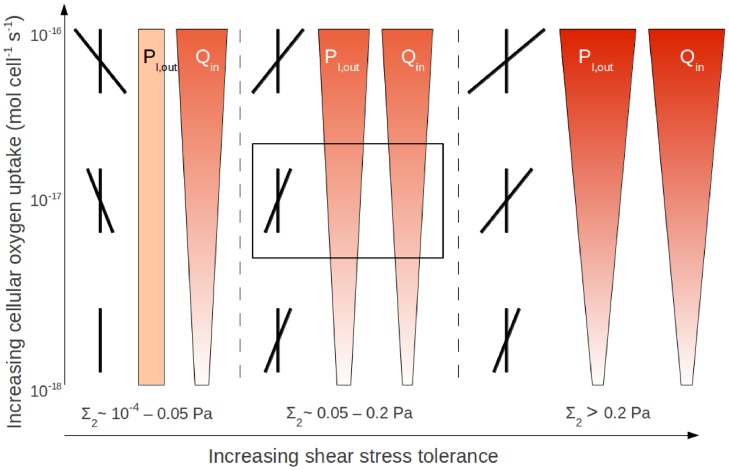
Optimal HFB operating conditions for proliferation of cell types with different oxygen uptake rates and shear stress tolerances. Wedge width and shading show relative values of flow rate, 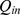, and outlet pressure, 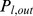, that give maximum cell yield for cells with different oxygen demands and shear tolerance; greater widths and darker shades representing greater values, up to 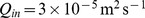 and 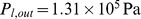. Angle of slanted line in crossed lines shows direction and degree of skew in position of initial aggregate distribution that gives maximum cell yield with optimal 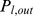 and 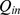 values; right slanting representing skew towards outlet and left slanting skew towards inlet end and a greater angle representing greater skew. Box marks optimal operating conditions for cells with oxygen uptake rate of 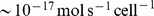 and shear tolerance of 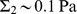 (see main text).

We have considered how the lumen inlet flow rate 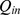 and outlet pressure 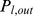 affect the growth of cell aggregates with different oxygen and shear stress sensitivites through their impact on the oxygen delivery to and shear stress on the cells. Increasing 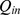 or 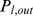 increases the transmembrane fluid flux, the increase being more pronounced for increments in 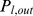 over the range of flow rates and pressures typically used in experiments. The increase in transmembrane fluid flux has two effects: more oxygen is advected through the membrane to the aggregates, and the horizontal flow in the ECS is stronger so the aggregates experience higher shear stress. The higher oxygen concentration increases the rate of aggregate growth, but the effect of the increased shear stress depends on the shear-sensitivity of the cells (i.e. the shear thresholds 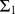 and 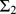, which mark the transitions to elevated proliferation rate and cell death) and the aggregate distribution, since the shear stress is non-uniform over the membrane. The shear stress increases from zero at the inlet end of the membrane to a maximum at the outlet end, which ranges between 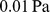 and 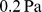 for the range of flow rates and outlet pressures considered (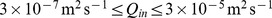 and 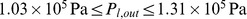 (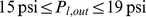)). Figure 10 summarises the operating conditions and basic seeding strategy that maximise the cell yield for cells with different oxygen uptake rates and shear stress tolerances. The optimal initial position of the aggregates is described in terms of the skew of the aggregate distribution towards the ECS outlet. For rat cardiomyocytes with an uptake rate of 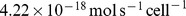 and shear tolerance of 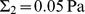, for example, the optimal operating conditions and aggregate seeding strategy are 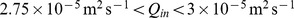 and 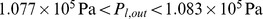 (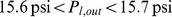) and an even distribution.

It is evident from Figure 10 that achieving the maximum cell yield requires a careful balance of the flow rate, outlet pressure and seeding distribution. For instance, the optimal seeding distribution of the aggregates changes from moving towards the outlet with increasing oxygen demand when 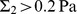 to moving towards the inlet when 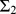 drops below 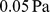. This is because the low shear tolerance of the cells prevents aggregates from growing near the outlet despite the improved oxygen delivery that can be achieved by seeding them there. The extent to which the cell seeding process can be controlled is thus an important issue. Indeed, the more random it is, the more likely aggregates are to form in regions with unfavorable growth conditions. This motivates further experimental studies to improve the control over the initial cell distribution achievable with seeding protocols. In the meantime, for random seeding of cells with typical oxygen demands, the cell yield can be optimised by using a flow rate and outlet pressure that ensure the shear stress is in the elevated proliferation rate range over as much of the membrane as possible. For example, from Figure 10 (see box) we can see that for cells with an oxygen uptake rate of the order of 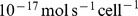 and a shear stress death threshold 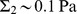 in an initial aggregate distribution skewed slightly towards the outlet, a flow rate of approximately 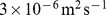 and an outlet pressure of 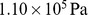 (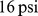) will ensure that the region of shear-stress-enhanced proliferation is as large as possible.

## Conclusions

We have developed a mathematical model of cell proliferation in a single-fibre HFB to optimise HFB operation for cell expansion. The model is used to investigate how the flow of culture medium through the HFB and the seeding distribution affect the subsequent growth of the cell population when it is mediated by oxygen delivery and fluid shear stress. By parameterising against cell-type specific data, the model can be used to determine the seeding distribution and values of the operating parameters—the lumen inlet flow rate and outlet pressure—that maximise the cell yield.

## Supporting Information

Table S1 **Dimensionless fluid and oxygen transport parameter values.**
(PDF)

Table S2 **Oxygen uptake rate data for different cell types grown in the extra-capillary space of hollow fibre bioreactors.**
(PDF)

Table S3 **Average cell diameters for different cell types cultured in 3D perfusion bioreactors.**
(PDF)

Table S4 **Half maximal oxygen uptake concentration, minimum oxygen concentration required to maintain cell viability, and inlet oxygen concentration for various cell types.**
(PDF)

Table S5 **Shear stress ranges used to culture different cell types in different perfusion bioreactors.**
(PDF)

Table S6 **Effects of different shear stress regimes on different cell types in perfusion bioreactors.**
(PDF)

File S1 **Model reduction, parameter estimation and numerical methods.** Section A: Analytical model reduction, Section B: Parameter Values, Section C: Solution of the reduced model.
(PDF)
