# Supplementary material for: Optimising Cell Aggregate Expansion in a Perfused Hollow Fibre Bioreactor via Mathematical Modelling
Source: PLoS One. 2014 Aug 26;9(8):e105813. doi: 10.1371/journal.pone.0105813 (PMC4144904; doi:10.1371/journal.pone.0105813)
Supplement: Table S1 — Dimensionless fluid and oxygen transport parameter values. (PDF) [file pone.0105813.s001.pdf]

Table S1. Dimensionless fluid and oxygen transport parameter values.

Parameter	Description	Typical Value(s)
$\epsilon = H/L$	lumen aspect ratio	2×10^{-3}
h_m	fibre outer radius	2
h_e	ECS outer radius	5
$\kappa_m = \epsilon^2 H^2 / k_m$	inverse membrane permeability	669
$p_{l,out} = \frac{\epsilon^2 L}{\mu U}(P_{l,out} - P_{atm})$	lumen outlet pressure	6.45 – 645
$k_{lo} = \mu K_{lo}/(\epsilon^3 L)$	permeability of membrane outer surface + cell aggregate	0.1
$k_{hi} = \mu K_{hi}/(\epsilon^3 L)$	permeability of membrane outer surface	1
$\epsilon^2 \mathrm{Pe}_l = \epsilon^2 UL/D_l$	reduced Péclet number in lumen	0.2 – 17.7
$\epsilon^2 \mathrm{Pe}_m = \epsilon^2 UL/D_m$	reduced Péclet number in membrane	2 – 177
$\epsilon^2 \mathrm{Pe}_e = \epsilon^2 UL/D_e$	reduced Péclet in ECS	0.2 – 17.7

From dimensional values in Table 1 in the main paper.
