# Supplementary material for: Optimising Cell Aggregate Expansion in a Perfused Hollow Fibre Bioreactor via Mathematical Modelling
Source: PLoS One. 2014 Aug 26;9(8):e105813. doi: 10.1371/journal.pone.0105813 (PMC4144904; doi:10.1371/journal.pone.0105813)
Supplement: Table S2 — Oxygen uptake rate data for different cell types grown in the extra-capillary space of hollow fibre bioreactors. (PDF) [file pone.0105813.s002.pdf]

Table S2. Oxygen uptake rate data for different cell types grown in the extra-capillary space of hollow fibre bioreactors.

Cell type	Cell density (cells m^{-3})	Maximal volumetric oxygen uptake rate (mol m^{-3} s^{-1})	Cellular oxygen uptake rate (mol s^{-1}cell^{-1})	Reference
Neonatal rat cardiomyocytes	10^{12}	2.64×10^{-3}	4.22×10^{-18} *	[1]
Primary rat hepatocytes	1.25×10^{13}	1.76×10^{-3}	2.82×10^{-18} *	[2]
Pancreatic βTC3 cells	2.8×10^{14}	6.37×10^{-3}	1.02×10^{-17} *	[3]
Chinese hamster lung H1 fibroblasts	10^{12}	3.9×10^{-5}	3.9×10^{-17}	[4]
Mouse hybridoma CR1606 cells	2×10^{14}	2.8×10^{-5}	2.8×10^{-17}	[5, 6]
Bovine chondrocytes	2×10^{14}	1.17×10^{-3}	1.86×10^{-18}	[7]
Human mesenchymal stem cells (hMSCs)	10^{12}	3.3×10^{-6}	3.3×10^{-18}	[8]
Rat hepatocytes	5×10^{12}	4.45×10^{-4}	8.9×10^{-17}	[9]
HepG2 hepatocytes	10^{13}	2.4×10^{-4}	2.4×10^{-17}	[10]
Porcine hepatocytes	2.5×10^{13}	8.25×10^{-4}	3.3×10^{-17}	[11]
Human foreskin fibroblasts (HFFs)	3.8×10^{12}	1.15×10^{-4}	3×10^{-17}	[12]

Adapted from [13]. For more data on oxygen uptake rates for hepatocytes see Table 1 in [14].
* Calculated by multiplying volumetric oxygen uptake rate by cell volume 1.6×10^{-15} m^3, assuming cells are spherical with diameter of 14.5µm (average of cell diameters in Table S3).

References

1. Radisic M, Deen W, Langer R, Vunjak-Novakovic G (2005) Mathematical model of oxygen distribution in engineered cardiac tissue with parallel channel array perfused with culture medium containing oxygen carriers. American Journal of Physiology-Heart and Circulatory Physiology 288: H1278–H1289.

2. Sullivan JP, Gordon JE, Bou-Akl T, Matthew HWT, Palmer AF (2007) Enhanced oxygen delivery to primary hepatocytes within a hollow fiber bioreactor facilitated via hemoglobin-based oxygen carriers. Artificial Cells, Blood Substitutes and Biotechnology 35: 585–606.

3. Tziampazis E, Sambanis A (1995) Tissue engineering of a bioartificial pancreas: modeling the cell environment and device function. Biotechnology Progress 11: 115–126.

4. Tharakan JP, Chau PC (1986) A radial flow hollow fiber bioreactor for the large-scale culture of mammalian cells. Biotechnology and Bioengineering 28: 329–342.

5. Piret JM, Cooney CL (1990) Mammalian cell and protein distributions in ultrafiltration hollow fiber bioreactors. Biotechnology and Bioengineering 36: 902–910.

6. Piret JM, Cooney CL (1991) Model of oxygen transport limitations in hollow fiber bioreactors. Biotechnology and Bioengineering 37: 80–92.

7. Obradovic B, Meldon JH, Freed LE, Vunjak-Novakovic G (2000) Glycosaminoglycan deposition in engineered cartilage: experiments and mathematical model. AIChE journal 46: 1860–1871.

8. Zhao F, Pathi P, Grayson W, Xing Q, Locke BR, et al. (2005) Effects of oxygen transport on 3-d human mesenchymal stem cell metabolic activity in perfusion and static cultures: Experiments and mathematical model. Biotechnology Progress 21: 1269–1280.

9. Shatford RA, Nyberg SL, Meier SJ, White JG, Payne WD, et al. (1992) Hepatocyte function in a hollow fiber bioreactor: a potential bioartificial liver. Journal of Surgical Research 53: 549–557.

10. Nyberg SL, Remmel RP, Mann HJ, Peshwa MV, Hu WS, et al. (1994) Primary hepatocytes outperform Hep G2 cells as the source of biotransformation functions in a bioartificial liver. Annals of Surgery 220: 59–67.

11. Custer L, Mullon CJ (1999) Oxygen delivery to and use by primary porcine hepatocytes in the HepatAssist 2000 system for extracorporeal treatment of patients in end-stage liver failure. Advances in Experimental Medicine and Biology. Springer, 261–271 pp.

12. Korin N, Bransky A, Dinnar U, Levenberg S (2007) A parametric study of human fibroblasts culture in a microchannel bioreactor. Lab on a Chip 7: 611–617.

13. Shipley RJ, Waters SL (2012) Fluid and mass transport modelling to drive the design of cell-packed hollow fibre bioreactors for tissue engineering applications. Mathematical Medicine and Biology 29: 329-359.

14. Patzer JF (2004) Oxygen consumption in a hollow fiber bioartificial liver—revisited. Artificial Organs 28: 83–98.
