# Supplementary material for: Optimising Cell Aggregate Expansion in a Perfused Hollow Fibre Bioreactor via Mathematical Modelling
Source: PLoS One. 2014 Aug 26;9(8):e105813. doi: 10.1371/journal.pone.0105813 (PMC4144904; doi:10.1371/journal.pone.0105813)
Supplement: Table S3 — Average cell diameters for different cell types cultured in 3D perfusion bioreactors. (PDF) [file pone.0105813.s003.pdf]

Table S3. Average cell diameters for different cell types cultured in 3D perfusion bioreactors.

Cell type	Cell diameter (µm)	Reference
Chinese hamster ovary (CHO)	12.8	[1]
NS1-derived mouse hybridoma	13.7	[2]
Human embryonic kidney (HEK293)	14.5	[3]
CHO	13.9	[4]
CHO	17.1	[5]

References

1. Seewöster T, Lehmann J (1997) Cell size distribution as a parameter for the predetermination of exponential growth during repeated batch cultivation of CHO cells. Biotechnology and Bioengineering 55: 793–797.

2. Hiller GW, Clark DS, Blanch HW (1993) Cell retention–chemostat studies of hybridoma cellsanalysis of hybridoma growth and metabolism in continuous suspension culture in serum-free medium. Biotechnology and Bioengineering 42: 185–195.

3. Kamen A, Henry O (2004) Development and optimization of an adenovirus production process. The Journal of Gene Medicine 6: S184–S192.

4. Hoesli CA, Luu M, Piret JM (2009) A novel alginate hollow fiber bioreactor process for cellular therapy applications. Biotechnology Progress 25: 1740–1751.

5. Clincke M, Zhang Y, Lindskog E, Walsh K, Chotteau Vt (2013) Very high density of CHO cells in perfusion by ATF or TFF in WAVE bioreactor. Part I. Effect of the cell density on the process. Biotechnology Progress 29: 768–777.
