# Supplementary material for: Optimising Cell Aggregate Expansion in a Perfused Hollow Fibre Bioreactor via Mathematical Modelling
Source: PLoS One. 2014 Aug 26;9(8):e105813. doi: 10.1371/journal.pone.0105813 (PMC4144904; doi:10.1371/journal.pone.0105813)
Supplement: Table S4 — Half maximal oxygen uptake concentration, minimum oxygen concentration required to maintain cell viability, and inlet oxygen concentration for various cell types. (PDF) [file pone.0105813.s004.pdf]

Table S4. Half maximal oxygen uptake concentration, minimum oxygen concentration required to maintain cell viability, and inlet oxygen concentration for various cell types.

Cell type	C_{in} (mol m^{-3})	$C_{1/2}$ (mol m^{-3})	C_0 (mol m^{-3})	Reference
Neonatal rat cardiomyocytes	0.22	6.9×10^{-3}	$6 \times 10^{-3} - 8 \times 10^{-2}$	[1,2]
Primary rat hepatocytes	0.22	6.24×10^{-3}	2.1×10^{-2}	[3,4]
Pancreatic βTC3 cells	0.22	1.0×10^{-2}	1.46×10^{-2}	[5,6]
Bovine chondrocytes	0.1	5.0×10^{-3}	$2.2 \times 10^{-3} - 1.32 \times 10^{-2}$	[7]
HFFs	0.19	2.1×10^{-3}	2.1×10^{-2}	[8]

Adapted from [9].

References

1. Radisic M, Deen W, Langer R, Vunjak-Novakovic G (2005) Mathematical model of oxygen distribution in engineered cardiac tissue with parallel channel array perfused with culture medium containing oxygen carriers. American Journal of Physiology-Heart and Circulatory Physiology 288: H1278–H1289.

2. Carrier RL, Papadaki M, Rupnick M, Schoen FJ, Bursac N, et al. (1999) Cardiac tissue engineering: cell seeding, cultivation parameters, and tissue construct characterization. Biotechnology and Bioengineering 64: 580–589.

3. Sullivan JP, Gordon JE, Bou-Akl T, Matthew HWT, Palmer AF (2007) Enhanced oxygen delivery to primary hepatocytes within a hollow fiber bioreactor facilitated via hemoglobin-based oxygen carriers. Artificial Cells, Blood Substitutes and Biotechnology 35: 585–606.

4. Consolo F, Fiore GB, Truscello S, Caronna M, Morbiducci U, et al. (2008) A computational model for the optimization of transport phenomena in a rotating hollow-fiber bioreactor for artificial liver. Tissue Engineering Part C: Methods 15: 41–55.

5. Tziampazis E, Sambanis A (1995) Tissue engineering of a bioartificial pancreas: modeling the cell environment and device function. Biotechnology Progress 11: 115–126.

6. Stabler CL, Fraker C, Pedraza E, Constantinidis I, Sambanis A (2009) Modeling and in vitro and in vivo characterization of a tissue engineered pancreatic substitute. Journal of Combinatorial Optimization 17: 54–73.

7. Fermor B, Christensen SE, Youn I, Cernanec JM, Davies CM, et al. (2007) Oxygen, nitric oxide and articular cartilage. European Cell Materials 13: 56–65.

8. Korin N, Bransky A, Dinnar U, Levenberg S (2007) A parametric study of human fibroblasts culture in a microchannel bioreactor. Lab on a Chip 7: 611–617.

9. Shipley RJ, Waters SL (2012) Fluid and mass transport modelling to drive the design of cell-packed hollow fibre bioreactors for tissue engineering applications. Mathematical Medicine and Biology 29: 329-359.
