# Supplementary material for: Optimising Cell Aggregate Expansion in a Perfused Hollow Fibre Bioreactor via Mathematical Modelling
Source: PLoS One. 2014 Aug 26;9(8):e105813. doi: 10.1371/journal.pone.0105813 (PMC4144904; doi:10.1371/journal.pone.0105813)
Supplement: Table S5 — Shear stress ranges used to culture different cell types in different perfusion bioreactors. (PDF) [file pone.0105813.s005.pdf]

Table S5. Shear stress ranges used to culture different cell types in different perfusion bioreactors.

Cell type	Bioreactor type	Shear stress range (Pa)	Duration	Reference
Human mesenchymal stem cells (hMSCs)	3D steady perfusion	1×10^{-5} – 1.2×10^{-4}	20 days	[1]
Bone marrow stromal cells (BMSCs)	2D parallel plate flow chamber	0.16	5 – 120 mins every other day 20 days	[2]
Rat BMSCs	2D radial flow device	0.036 – 0.27	30 mins every other day for 13 days	[3]
Rat BMSCs	3D steady perfusion	0.1 – 0.2	16 days	[4]
Human bone marrow MSCs	3D perfusion	0.005 – 0.015	28 days	[5]
Fetal calf endothelial cells	2D rotating dish	0.03 – 0.17	24 hrs	[6]
Fibroblasts	3D perfusion	0.1 – 2	72 hrs	[7]
Bovine aortic endothelial cells	2D parallel plate flow chamber	1.2	5 mins-16 hrs	[8]
Rat calvarial osteoblasts & human umbilical vein endothelial cells & baby hamster kidney fibroblasts	2D parallel flow chamber	0.01 – 3.5	15 mins	[9]
Rat BMSCs	3D steady perfusion	0.005	4 – 16 days	[10]
Mouse osteosarcoma K8	3D steady perfusion	1.57×10^{-4}	21 days	[11]
Mouse MGZ5 embryonic stem cells	2D parallel plate flow chamber	0.15 – 1	24-72 hrs	[12]
Human hepatocytes	rotating HFMB	0.05	12 days	[13]
Rat cardiomyoctyes	parallel micro-channel array	0.1	3 days	[14]
HFFs	micro-channel flat plate	0.02	14 days	[15]
Mouse calvarial osteoblasts MC3T3-E1	3D micro-channel array	0.005	7 days	[16]

References

1. Zhao F, Chella R, Ma T (2007) Effects of shear stress on 3-D human mesenchymal stem cell construct development in a perfusion bioreactor system: Experiments and hydrodynamic modeling. Biotechnology and Bioengineering 96: 584–595.

2. Kreke MR, Huckle WR, Goldstein AS (2005) Fluid flow stimulates expression of osteopontin and bone sialoprotein by bone marrow stromal cells in a temporally dependent manner. Bone 36: 1047–1055.

3. Kreke MR, Goldstein AS (2004) Hydrodynamic shear stimulates osteocalcin expression but not proliferation of bone marrow stromal cells. Tissue Engineering 10: 780–788.

4. Bancroft GN, Sikavitsas VI, van den Dolder J, Sheffield TL, Ambrose CG, et al. (2002) Fluid flow increases mineralized matrix deposition in 3D perfusion culture of marrow stromal osteoblasts in a dose-dependent manner. Proceedings of the National Academy of Sciences 99: 12600–12605.

5. Li D, Tang T, Lu J, Dai K (2009) Effects of flow shear stress and mass transport on the construction of a large-scale tissue-engineered bone in a perfusion bioreactor. Tissue Engineering Part A 15: 2773–2783.

6. Ando J, Nomura H, Kamiya A (1987) The effect of fluid shear stress on the migration and proliferation of cultured endothelial cells. Microvascular Research 33: 62–70.

7. Lesman A, Blinder Y, Levenberg S (2010) Modeling of flow-induced shear stress applied on 3D cellular scaffolds: Implications for vascular tissue engineering. Biotechnology and Bioengineering 105: 645–654.

8. Tzima E, Irani-Tehrani M, Kiosses WB, Dejana E, Schultz DA, et al. (2005) A mechanosensory complex that mediates the endothelial cell response to fluid shear stress. Nature 437: 426–431.

9. Reich KM, Gay CV, Frangos JA (1990) Fluid shear stress as a mediator of osteoblast cyclic adenosine monophosphate production. Journal of Cellular Physiology 143: 100–104.

10. Sikavitsas VI, Bancroft GN, Lemoine JJ, Liebschner MAK, Dauner M, et al. (2005) Flow perfusion enhances the calcified matrix deposition of marrow stromal cells in biodegradable nonwoven fiber mesh scaffolds. Annals of Biomedical Engineering 33: 63–70.

11. McGarry JG, Klein-Nulend J, Mullender MG, Prendergast PJ (2005) A comparison of strain and fluid shear stress in stimulating bone cell responsesa computational and experimental study. Journal of the Federation of American Societies for Experimental Biology 19: 482–484.

12. Yamamoto K, Sokabe T, Watabe T, Miyazono K, Yamashita JK, et al. (2005) Fluid shear stress induces differentiation of Flk-1-positive embryonic stem cells into vascular endothelial cells in vitro. American Journal of Physiology-Heart and Circulatory Physiology 288: H1915–H1924.

13. Consolo F, Fiore GB, Truscello S, Caronna M, Morbiducci U, et al. (2008) A computational model for the optimization of transport phenomena in a rotating hollow-fiber bioreactor for artificial liver. Tissue Engineering Part C: Methods 15: 41–55.

14. Radisic M, Deen W, Langer R, Vunjak-Novakovic G (2005) Mathematical model of oxygen distribution in engineered cardiac tissue with parallel channel array perfused with culture medium containing oxygen carriers. American Journal of Physiology-Heart and Circulatory Physiology 288: H1278–H1289.

15. Korin N, Bransky A, Dinnar U, Levenberg S (2007) A parametric study of human fibroblasts culture in a microchannel bioreactor. Lab on a Chip 7: 611–617.

16. Leclerc E, David B, Griscom L, Lepioufle B, Fujii T, et al. (2006) Study of osteoblastic cells in a microfluidic environment. Biomaterials 27: 586–595.
