# Supplementary material for: Optimising Cell Aggregate Expansion in a Perfused Hollow Fibre Bioreactor via Mathematical Modelling
Source: PLoS One. 2014 Aug 26;9(8):e105813. doi: 10.1371/journal.pone.0105813 (PMC4144904; doi:10.1371/journal.pone.0105813)
Supplement: Table S6 — Effects of different shear stress regimes on different cell types in perfusion bioreactors. (PDF) [file pone.0105813.s006.pdf]

Table S6. Effects of different shear stress regimes on different cell types in perfusion bioreactors.

Cell type	Bioreactor type	Shear stress threshold	Effect	Reference
Endothelial	2D parallel plate laminar flow chamber	2.3 Pa for 1 hrs	no adverse effect	[1]
Rat hepatocytes	micro-channel flat plate	0.1 – 1 Pa for 12 hrs	no adverse affect	[1]
Leukocytes	stirred-flask	0.5 – 10 Pa for 12 hrs	adherent cells detached from surfaces	[1]
Human osteoblasts	2D parallel plate flow chamber	2 Pa for 30 mins	increased proliferation	[2]
Human bone marrow stromal cells	2D parallel plate flow chamber	1.2×10^{-3} Pa for 10 days	slower proliferation	[3]
Mouse MGZ5 embryonic stem cells	2D parallel plate flow chamber	0.15 – 1 Pa for 24-72 hrs	increased proliferation	[4]
Human mesenchymal stem cells (hMSCs)	3D steady perfusion	1.2×10^{-4} Pa for 20 days	increased proliferation	[5]
Human hepatocytes	rotating HFMB	0.5 – 2 Pa for 12 days	specific functions of liver cells compromised leading to toxicity	[6]
		> 2 Pa	cell death	[6]
Rat cardiomyoctyes	parallel micro-channel array	> 0.16 Pa	cell damage and death	[7]
HFFs	micro-channel flat plate	> 0.03 Pa for 2 days	cell detachment	[8]
Mouse calvarial osteoblasts MC3T3-E1	3D micro-channel array	0.035 Pa for 2 days	cell detachment	[9]

References

1. Zeng Y, Lee TS, Yu P, Roy P, Low HT (2006) Mass transport and shear stress in a microchannel bioreactor: numerical simulation and dynamic similarity. Journal of Biomechanical Engineering 128: 185–193.

2. Kapur S, Baylink DJ, Lau K, William Lau KH (2003) Fluid flow shear stress stimulates human osteoblast proliferation and differentiation through multiple interacting and competing signal transduction pathways. Bone 32: 241–251.

3. Scaglione S, Wendt D, Miggino S, Papadimitropoulos A, Fato M, et al. (2008) Effects of fluid flow and calcium phosphate coating on human bone marrow stromal cells cultured in a defined 2D model system. Journal of Biomedical Materials Research Part A 86: 411–419.

4. Yamamoto K, Sokabe T, Watabe T, Miyazono K, Yamashita JK, et al. (2005) Fluid shear stress induces differentiation of Flk-1-positive embryonic stem cells into vascular endothelial cells in vitro. American Journal of Physiology-Heart and Circulatory Physiology 288: H1915–H1924.

5. Zhao F, Chella R, Ma T (2007) Effects of shear stress on 3-D human mesenchymal stem cell construct development in a perfusion bioreactor system: Experiments and hydrodynamic modeling. Biotechnology and Bioengineering 96: 584–595.

6. Consolo F, Fiore GB, Truscello S, Caronna M, Morbiducci U, et al. (2008) A computational model for the optimization of transport phenomena in a rotating hollow-fiber bioreactor for artificial liver. Tissue Engineering Part C: Methods 15: 41–55.

7. Radisic M, Deen W, Langer R, Vunjak-Novakovic G (2005) Mathematical model of oxygen distribution in engineered cardiac tissue with parallel channel array perfused with culture medium containing oxygen carriers. American Journal of Physiology-Heart and Circulatory Physiology 288: H1278–H1289.

8. Korin N, Bransky A, Dinnar U, Levenberg S (2007) A parametric study of human fibroblasts culture in a microchannel bioreactor. Lab on a Chip 7: 611–617.

9. Leclerc E, David B, Griscom L, Lepioufle B, Fujii T, et al. (2006) Study of osteoblastic cells in a microfluidic environment. Biomaterials 27: 586–595.
