# Supplementary material for: Optimising Cell Aggregate Expansion in a Perfused Hollow Fibre Bioreactor via Mathematical Modelling
Source: PLoS One. 2014 Aug 26;9(8):e105813. doi: 10.1371/journal.pone.0105813 (PMC4144904; doi:10.1371/journal.pone.0105813)
Supplement: File S1 — Model reduction, parameter estimation and numerical methods. Section A: Analytical model reduction, Section B: Parameter Values, Section C: Solution of the reduced model. (PDF) [file pone.0105813.s007.pdf]

Model Reduction, Parameter Estimation and Numerical Methods

A Analytical model reduction

The full model for the fluid flow, oxygen transport and aggregate growth in the HFB is given by Equations (1)–(23). We nondimensionalise these equations using the following scalings

$$x = L\hat{x}, \quad y = H\hat{y}, \quad u_i = U\hat{u}_i, \quad v_i = \epsilon U\hat{v}_i, \quad t = \frac{2}{C_{in}A_1}\hat{t}, \quad c_i = C_{in}\hat{c}_i \quad i = l, m, e,$$
$$p_i = P_{atm} + \frac{\mu U}{\epsilon^2 L}\hat{p}_i, \quad i = l, e, \quad p_m = P_{atm}/\phi + \frac{\epsilon^2 \mu U L}{k_m}\hat{p}_m, \quad \boldsymbol{\sigma}_i = \frac{\mu U}{\epsilon^2 L}\hat{\boldsymbol{\sigma}}_i, \quad i = l, e,$$
$$\boldsymbol{\sigma}_m = \frac{\epsilon^2 \mu U L}{k_m}\hat{\boldsymbol{\sigma}}_m, \quad H_m = Hh_m, \quad H_e = Hh_e, \quad p_{l,out} = \frac{\epsilon^2 L}{\mu U}(P_{l,out} - P_{atm}), \tag{S1}$$

where $U = Q_{in}/H$ and $\epsilon = H/L \ll 1$. Henceforth, we omit hats on dimensionless variables for convenience. The timescale is chosen as that for aggregate growth, $2/(C_{in}A_1)$, and represents the time taken for the cell population to double at high oxygen concentration ($c_e > C_2$) and low shear stress ($\sigma_{e,xy} < \Sigma_1$) (see Section B). On this timescale, the fluid and oxygen transport problems are quasi-steady. The pressure scale in the lumen and ECS ($\mu U/(\epsilon^2 L)$) is chosen to balance the axial pressure gradient with the tranverse viscous terms (in Equation (1)), and that in the membrane ($\epsilon^2 \mu U L/k_m$) to balance the velocity components in the continuity of flux condition (in Equation (3)). In the following sections we have exploited the fact that $\epsilon = H/L = 2 \times 10^{-3} \ll 1$ to simplify the governing equations by neglecting terms of $\mathcal{O}(\epsilon^2)$. The equations in Sections A.1–A.3 and C.1 below are presented at leading order in ϵ unless otherwise stated.

A.1 Fluid transport

The flow equations in the lumen, ECS and membrane (from Equation (1)) are

$$\frac{\partial u_i}{\partial x} + \frac{\partial v_i}{\partial y} = 0, \quad i = l, m, e, \tag{S2}$$

$$\frac{\partial^2 u_i}{\partial y^2} = \frac{\partial p_i}{\partial x}, \quad \frac{\partial p_i}{\partial y} = 0 \quad \Rightarrow \quad p_i = p_i(x,t), \quad i = l, e, \tag{S3}$$

$$u_m = 0,\; v_m = -\frac{\partial p_m}{\partial y}. \tag{S4}$$

On the lumen-membrane interface, the fluid flux continuity condition (from Equation (3)) is

$$v_l = \phi v_m \quad \text{on } y = 1. \tag{S5}$$

The dimensionless fluid stress tensors in the lumen, ECS and membrane are

$$\boldsymbol{\sigma}_i = \begin{pmatrix} \sigma_{i,xx} & \sigma_{i,xy} \\ \sigma_{i,xy} & \sigma_{i,yy} \end{pmatrix} = \begin{pmatrix} -p_i + 2\epsilon^2 \frac{\partial u_i}{\partial x} & \epsilon \frac{\partial u_i}{\partial y} + \epsilon^3 \frac{\partial v_i}{\partial x} \\ \epsilon \frac{\partial u_i}{\partial y} + \epsilon^3 \frac{\partial v_i}{\partial x} & -p_i + 2\epsilon^2 \frac{\partial v_i}{\partial y} \end{pmatrix}, \quad i = l, e, \quad \boldsymbol{\sigma}_m = -p_m \boldsymbol{I}. \tag{S6}$$

Hence, the normal stress boundary condition in (3) reduces to

$$p_l = \phi \kappa_m p_m \quad \text{on } y = 1, \tag{S7}$$

where $\kappa_m := \epsilon^2 H^2 / k_m$ is a dimensionless parameter representing the permeability of the membrane. The boundary condition for the fluid flux through the outer surface of the membrane (Equation (5)) reduces to Starling's equation

$$\phi v_m = v_e = k(x,t)(\phi \kappa_m p_m - p_e) \quad \text{on } y = h_m, \tag{S8}$$

where the dimensionless membrane outer surface permeability function, $k(x,t)$, is given by

$$k(x,t) = \frac{\mu}{\epsilon^3 L} K(Lx, 2/(C_{in} A_1)t) = k_{lo} I_c(x,t) + k_{hi}(1 - I_c(x,t)), \tag{S9}$$

and $k_{lo} = \mu K_{lo}/(\epsilon^3 L)$ and $k_{hi} = \mu K_{hi}/(\epsilon^3 L)$ are the dimensionless aggregate and membrane outer surface permeabilities, assumed to be $\mathcal{O}(1)$ to retain as many physical effects in the model at leading order as possible, and

$$I_c(x,t) = \begin{cases} 1 & \text{for } x \text{ in an aggregate at time } t, \\ 0 & \text{for } x \text{ not in an aggregate at time } t, \end{cases} \tag{S10}$$

is the cell indicator function. The pressure scaling in the membrane gives $u_m = -\epsilon^2 \frac{\partial p_m}{\partial x} = \mathcal{O}(\epsilon^2)$, so the no-slip conditions on the inner and outer surface of the membrane (from Equation (6)) are

$$u_l = 0 \quad \text{on } y = 1, \quad u_e = 0 \quad \text{on } y = h_m. \tag{S11}$$

The dimensionless symmetry, no-slip and no-flux boundary conditions on the outer boundaries (from Equations (7), (8)) are

$$v_l = 0, \ \frac{\partial u_l}{\partial y} = 0 \quad \text{on } y = 0, \tag{S12}$$

$$u_e = 0, \ v_e = 0 \quad \text{on } y = h_e, \tag{S13}$$

$$u_e = 0 \quad \text{on } x = 0, \, h_m < y < h_e. \tag{S14}$$

The conditions at the inlet and outlets (Equations (10)–(12)) become

$$\int_0^1 u_l|_{x=0} \, \mathrm{d}y = 1, \tag{S15}$$

$$p_l = p_{l,out} \quad \text{on } x = 1, \, 0 < y < 1, \tag{S16}$$

$$p_e = 0 \quad \text{on } x = 1, \, h_m < y < h_e. \tag{S17}$$

A.2 Oxygen transport

The leading-order dimensionless oxygen transport equations are

$$\epsilon^2 \mathrm{Pe}_i \left(u_i \frac{\partial c_i}{\partial x} + v_i \frac{\partial c_i}{\partial y} \right) = \frac{\partial^2 c_i}{\partial y^2}, \quad i = l, m, e, \tag{S18}$$

where $\epsilon^2 \mathrm{Pe}_i = \epsilon^2 UL/D_i = \mathcal{O}(1)$ $(i = l, m, e)$ are the reduced Péclet numbers in the different regions (as defined in the Parameter Values Section in the main paper). The conditions on the interfaces between the membrane and the lumen and ECS (Equations (14), (15)) become

$$c_l = c_m, \quad \frac{D_l}{D_m} \frac{\partial c_l}{\partial y} = \frac{\partial c_m}{\partial y} \quad \text{on } y = 1, \tag{S19}$$

$$c_m = c_e, \quad \frac{\partial c_e}{\partial y} - \frac{D_m}{D_e} \frac{\partial c_m}{\partial y} = \gamma I_c(x,t) \frac{c_e}{c_{1/2} + c_e} \quad \text{on } y = h_m, \tag{S20}$$

where D_l/D_m is assumed to be $\mathcal{O}(1)$ (as for the values in Table B in the main paper), $\gamma = H\Gamma/(D_e C_{in})$ is the second Damköhler number (introduced in the Parameter Values Section in the main paper), and $c_{1/2} = C_{1/2}/C_{in}$ is the dimensionless half maximal uptake flux concentration. The dimensionless forms of boundary conditions (16)–(20) are

$$c_l = 1 \quad \text{on } x = 0,\ 0 < y < 1, \quad \frac{\partial c_l}{\partial x} = 0 \quad \text{on } x = 1,\ 0 < y < 1, \quad \frac{\partial c_l}{\partial y} = 0 \quad \text{on } y = 0, \tag{S21}$$

$$\frac{\partial c_m}{\partial x} = 0 \quad \text{on } x = 0, 1,\ 1 < y < h_m, \tag{S22}$$

$$\frac{\partial c_e}{\partial x} = 0 \quad \text{on } x = 0, 1,\ h_m < y < h_e, \quad \frac{\partial c_e}{\partial y} = 0 \quad \text{on } y = h_e. \tag{S23}$$

A.3 Cell aggregate growth

From the dimensionless fluid stress tensor in (S6) we can see that the dominant component of the shear stress along the membrane in the ECS,

$$\sigma_{e,xy}|_{y=h_m} = \left(\epsilon \frac{\partial u_e}{\partial y} + \epsilon^3 \frac{\partial v_e}{\partial x} \right) \bigg|_{y=h_m},$$

is $\epsilon \frac{\partial u_e}{\partial y}\Big|_{y=h_m}$, so we approximate the shear stress by this component. The dimensionless growth equation (from Equation (23)) is then given by

$$\frac{\mathrm{d}x_{2j}}{\mathrm{d}t} = -\frac{\mathrm{d}x_{2j-1}}{\mathrm{d}t}$$
$$= \int_{x_{2j-1}(t)}^{x_{2j}(t)} \left(g_p\left(c_e(x, H_m, t), \frac{\partial u_e}{\partial y}(x, H_m, t)\right) - g_d\left(c_e(x, H_m, t), \frac{\partial u_e}{\partial y}(x, H_m, t)\right)\right) \mathrm{d}x, \quad \text{(S24)}$$

where

$$g_p\left(c_e, \frac{\partial u_e}{\partial y}\right) = ((c_e - c_1)\mathrm{H}(c_e - c_1)\mathrm{H}(c_2 - c_e) + (c_2 - c_1)\mathrm{H}(c_e - c_2))$$
$$\times \left(\mathrm{H}\left(\sigma_1 - \frac{\partial u_e}{\partial y}\right) + \alpha \mathrm{H}\left(\frac{\partial u_e}{\partial y} - \sigma_1\right)\mathrm{H}\left(\sigma_2 - \frac{\partial u_e}{\partial y}\right)\right) \quad \text{(S25)}$$
$$g_d\left(c_e, \frac{\partial u_e}{\partial y}\right) = \beta_1 \mathrm{H}(c_0 - c_e) + \beta_2 \mathrm{H}\left(\frac{\partial u_e}{\partial y} - \sigma_2\right) + \beta_3 \mathrm{H}(c_0 - c_e)\mathrm{H}\left(\frac{\partial u_e}{\partial y} - \sigma_2\right), \quad \text{(S26)}$$

with $c_k = C_k/C_{in},\ k = 0, 1, 2$ the dimensionless concentration thresholds for cell survival, proliferation and maximal proliferation; $\sigma_k = \epsilon L \Sigma_k/(\mu U),\ k = 1, 2$ the dimensionless shear stress thresholds for faster proliferation and cell death; $\alpha = A_2/A_1 > 1$ the faster growth rate for intermediate shear stress relative to the baseline growth rate for low shear stress; and $\beta_k = B_0 B_k/(C_{in} A_1),\ k = 1, 2$ and $\beta_3 = B_0 B_1 B_2/(C_{in} A_1)$ the concentration and shear-stress dependent shortening rates relative to the baseline growth rate.

B Parameter values

B.1 Fluid and oxygen transport parameters

Typical values of the dimensional fluid and oxygen transport parameters are given in Table 1 in the main paper. Corresponding dimensionless parameters are given in Table S1. Values for the inlet oxygen concentration, oxygen uptake and threshold parameters are cell-type dependent. Values for the maximal oxygen uptake rates of different cell types are given in Table S2. The maximal volumetric uptake rate varies considerably with cell type and cell density. We assume

that for a given cell type the cell density in the aggregates is constant, even as they grow, so that the maximal uptake flux Γ is constant. The cellular oxygen uptake rate (the number of moles of oxygen taken up by one cell per second) lies in the range 1.86×10^{-18} – $8.9\times10^{-17}\,\mathrm{mol\,s^{-1}\,cell^{-1}}$. The order of magnitude range of Γ is estimated by multiplying the cellular oxygen uptake rate by the range of cell surface densities observed for Chinese hamster lung fibroblasts cultured for 12 days in a HFB by Tharakan and Chau [1]: 5×10^{8} – $7.3\times10^{10}\,\mathrm{cells\,m^{-2}}$,

$$\begin{aligned}\Gamma &= \text{cellular oxygen uptake rate} \times \text{cell surface density}\\ &= (1.86\times10^{-18} - 8.9\times10^{-17}\,\mathrm{mol\,s^{-1}\,cell^{-1}}) \;\times\; (5\times10^{8} - 7.3\times10^{10}\,\mathrm{cells\,m^{-2}})\\ &= 9.3\times10^{-10} - 6.5\times10^{-6}\,\mathrm{mol\,m^{-2}\,s^{-1}}.\end{aligned}$$

This agrees well with the estimate of the maximal oxygen uptake flux of human foreskin fibroblasts of $1.5\times10^{-8}\,\mathrm{mol\,m^{-2}\,s^{-1}}$ used by Korin et al. [2]. The corresponding range for γ is

$$\gamma = \frac{H\Gamma}{D_e C_{in}} = \frac{2\times10^{-4}}{3\times10^{-9}\times0.22}\Gamma = 2.8\times10^{-4} - 2.0.$$

Given that the reduced model is only valid when γ is order 1, it is not valid for cells with uptake rates at the lower end of the 1.86×10^{-18} – $8.9\times10^{-17}\,\mathrm{mol\,s^{-1}\,cell^{-1}}$ range unless they are seeded at high density ($\geq 7\times10^{10}\,\mathrm{cells\,m^{-2}}$).

B.2 Estimates of seeding and growth parameters

Using experimental data we obtain estimates of a typical initial cell density and aggregate elongation rate for the model setup.

B.2.1 Initial cell density

Ellis and Chaudhuri [3] seeded an 8 ml inoculum of 500,000 cells (equating to a cell concentration of $6.25\times10^{10}\,\mathrm{cells\,m^{-3}}$) onto 10 poly(lactic-co-glycolic) acid fibres of similar dimensions to those in Table 1 in the main paper. For a dynamic seeding protocol on a rotating mixer, they found

that on average 65% of the cells in the seeding mixture attached to the fibres. This gives an estimate of the initial cell density via

$$\begin{aligned}\text{no. of cells attached to each fibre} &= \text{no. of cells in inoculum/no. of fibres} \times \text{seeding efficiency}\\ &= 500,000/10 \times 0.65 = 32,500,\\ \text{surface area of fibre} &= 2\pi H_m L = 2\pi(4 \times 10^{-4}) \times 0.1 = 2.51\,\text{cm}^2,\\ \text{initial cell density} &= \text{no. of cells/fibre surface area}\\ &= 32,500/2.51 \approx 13,000\,\text{cells/cm}^2.\end{aligned}$$

When attached to the membrane, a typical cell has a diameter of 40 μm [2], so, if we assume that the cells are circular in cross-section parallel to the surface, the fraction of the membrane surface covered by the cells is

$$\begin{aligned}\text{fraction of membrane surface covered} &= \text{total area covered by cells/membrane surface area}\\ &= \text{no. of cells} \times \text{cell area/membrane surface area}\\ &= \text{cell density} \times \text{cell area}\\ &= 13,000 \times \pi(20 \times 10^{-4})^2 = 0.16.\end{aligned}$$

Assuming that the percentage coverage is the same in 1D as in 2D, i.e. 16% of the line along the outer surface of the membrane in our model is covered by cells, the initial number of cells on the line is approximately

$$\begin{aligned}\text{number of cells on membrane line segment} &= \text{length covered by cells/cell diameter}\\ &= (1.6 \times 10^{-2})/(4 \times 10^{-5}) = 400.\end{aligned}$$

This number corresponds to the cell concentration in the inoculum and seeding efficiency in [3]. For our simulations, we assume that 25% of the membrane is initially covered by cells (i.e. that

the initial total aggregate length is 2.5 cm, equivalent to 625 cells), corresponding to a higher concentration of cells in the inoculum or higher seeding efficiency or both.

B.2.2 Aggregate growth rate

We estimate the baseline aggregate growth rate, A_1, for the case in which the concentration and shear stress at the aggregates are constant and $c > C_2$, $\sigma_{e,xy} < \Sigma_1$, so that there is no cell death and the aggregate growth rate is proportional to the aggregate length, with $A_1(C_2 - C_1)$ as the constant of proportionality. That is, if L_i is the length of the ith aggregate, then

$$\frac{\mathrm{d}L_i}{\mathrm{d}t} = A_1(C_2 - C_1)L_i, \quad i = 1, \dots, N, \quad \Rightarrow \quad L_i(t) = L_i(0)e^{A_1(C_2-C_1)t}.$$

So, if we assume that the aggregate length doubles when the population doubles and there are ~ 1.5 population doublings in 7 days [3], we can use the fact that $C_2 - C_1 < C_{in}$ to calculate a lower bound for A_1

$$A_1 = \frac{\ln \frac{L_i}{L_i(0)}}{(C_2 - C_1)t} > \frac{1.5 \ln 2}{0.22 \times 7} = 0.68\,\mathrm{mol}^{-1}\,\mathrm{m}^3\,\mathrm{day}^{-1} = 7.8 \times 10^{-6}\,\mathrm{mol}^{-1}\,\mathrm{m}^3\,\mathrm{s}^{-1}.$$

This means that the aggregate elongation timescale is $2/(A_1 C_{in}) \sim 13.5\,\mathrm{days} = 1.2 \times 10^6\,\mathrm{s}$, i.e. is much longer than the fluid or oxygen transport timescales as assumed.

As described above, the functional form we adopt for the dependence of the growth on the oxygen concentration is an approximation of a sigmoidal relationship. Experimental data with which we can estimate the concentration thresholds for linear dependence of the growth rate on the concentration and the maximal growth rate, C_1 and C_2, is not readily available. Hence, we will pick different values of the concentration thresholds in the ranges $C_1 = 0.05 - 0.1\,\mathrm{mol}\,\mathrm{m}^{-3}$ and $C_2 = 0.18 - 0.21\,\mathrm{mol}\,\mathrm{m}^{-3}$, such that the range over which the growth rate is not constant (C_1 to C_2) is large.

Various shear stress regimes used to culture different cell types in perfusion bioreactors are given in Table S5. It is evident that the shear stresses used vary hugely depending on the cell

type, bioreactor and culture period. However, the shear stress regimes tend to fall into two broad categories: short-term, often intermittent, exposure (24 – 72 hrs) of 2D cell cultures to high shear stresses (0.01 – 2 Pa); and long-term steady exposure (7 – 21 days) of 3D cultures to much lower shear stresses (1×10^{-5} – 0.2 Pa). The latter category is more appropriate to the setup considered here and the shear stresses on the aggregates fall in the same range (see Results Section). In their respective reviews of the influence of shear stress on osteogenic cell lines, Yeatts and Fisher [4] and McCoy and O'Brien [5] identified the optimum shear stress ranges for 3D culture to be 0.005 – 0.1 Pa and 0.01 – 0.05 Pa. We use these ranges as a rough guide for the shear stress threshold for increased proliferation, Σ_1, considering values in the range $\Sigma_1 = 0.01 - 0.05$ Pa. Table S6 lists the effects of various shear stress regimes on different cell types. The data suggests that, while endothelial cells and osteoblasts may be tolerant to short-term exposure to shear stresses in excess of 2 Pa in parallel plate flow chambers, cells cultured for longer periods in microfluidic devices can be damaged by much lower shear stresses (0.03–0.16 Pa). For the shear stress threshold for cell death, Σ_2, we will use values in the range 0.05 – 0.16 Pa, appropriate to the cell types we consider.

C Solution of the reduced model

Below we describe how we solve the reduced model for the fluid flow, oxygen transport and aggregate growth given by Equations (S2)–(S17), (S18)–(S23) and (S24)–(S26).

Since the fluid and oxygen transport problems are quasi-steady they can be solved at any point in time for a given cell aggregate distribution. In theory, therefore, the only initial condition required to solve the aggregate growth problem is the initial aggregate distribution. However, in practice an initial condition for the oxygen concentration is also required as a consequence of the nonlinear uptake term and the numerical scheme (see Section C.3). The cell indicator function can then be evaluated from the initial aggregate distribution as described in Section C.2.

C.1 Fluid transport

First we derive the governing PDE and boundary conditions for the lumen pressure. We start by integrating the leading order fluid transport equations (Equations (S2)–(S4)) subject to the boundary conditions (S5)–(S17). The velocity components in each region are given by

$$u_l = \frac{1}{2}\frac{\partial p_l}{\partial x}(y^2-1), \quad v_l = \frac{1}{6}\frac{\partial^2 p_l}{\partial x^2}y(3-y^2), \tag{S27}$$

$$u_m = 0, \quad v_m = \frac{1}{3\phi}\frac{\partial^2 p_l}{\partial x^2}, \tag{S28}$$

$$u_e = \frac{1}{2}\frac{\partial p_e}{\partial x}(y-h_m)(y-h_e), \quad v_e = -\frac{1}{12}\frac{\partial^2 p_e}{\partial x^2}(y-h_e)^2(2y-3h_m+h_e), \tag{S29}$$

i.e. the flow in the lumen and ECS has a Poiseuille profile.

The pressures in the membrane and ECS are

$$p_m = \frac{1}{\phi}\left(\frac{p_l}{\kappa_m} - \frac{1}{3}\frac{\partial^2 p_l}{\partial x^2}(y-1)\right), \quad p_e = \frac{1}{(h_e-h_m)^3}(12q_{in}(1-x) - 4(p_l - p_{l,out})). \tag{S30}$$

Starling's equation on the interface between the membrane and the ECS (S8) gives the following second order PDE for p_l

$$\frac{1}{3}(1+\kappa_m(h_m-1)k(x,t))\frac{\partial^2 p_l}{\partial x^2} - \left(1+\frac{4}{(h_e-h_m)^3}\right)k(x,t)p_l = -\frac{k(x,t)}{(h_e-h_m)^3}(12(1-x)+4p_{l,out}), \tag{S31}$$

and (S15) and (S16) supply the boundary conditions for p_l at the inlet and outlet

$$\frac{\partial p_l}{\partial x}(0,t) = -3, \quad p_l(1,t) = p_{l,out}. \tag{S32}$$

The leading order system of equations for the fluid flow, oxygen transport and aggregate growth is thus given by Equations (S31), (S9), (S32), (S27)–(S29), (S18), (S19)–(S23), and (S24). We solve this system numerically as described in Section C.3. First, however, we describe the issues associated with the discrete definition of the cell indicator function, $I_c(x,t)$, in equation (S10) and how we use a smoothed version to overcome them.

C.2 Smoothed cell indicator function

With $I_c(x,t)$ as defined in (S10), the permeability of the outer surface of the membrane, $k(x,t)$, and oxygen uptake, $\gamma I_c(x,t)c_e/(c_{1/2}+c_e)$, are discontinuous at the ends of the cell aggregates. So in order to solve the boundary value problem for p_l given by (S31) and (S32) we would need to specify two boundary conditions at each end of each cell aggregate. The natural boundary conditions to use would be continuity of pressure and flux. However, a more serious issue with using the piecewise continuous I_c in (S10) is that $\frac{\partial^2 p_l}{\partial x^2}$, and therefore the vertical velocity components, are discontinuous at the ends of the cell aggregates.

To circumvent this problem, we use a smoothed version of the indicator function that is continuous at the ends of the cell aggregates to represent the membrane outer surface permeability and oxygen uptake. For N cell aggregates with ends at $x_i(t)$ $(i = 1, \ldots, 2N)$, the smoothed indicator function is

$$I_c(x,t) = \sum_{j=1}^{N} \frac{1}{2}(\tanh(a(x - x_{2j-1}(t))) + \tanh(a(x_{2j}(t) - x))), \tag{S33}$$

where the constant a determines the sharpness of the transition between the values of the permeability and uptake inside and outside the cell aggregate (Figure S1). The higher the value of a, the sharper the transition in the indicator function at the ends of the aggregates and the more closely it approximates the discrete cell indicator function. Defined in this way, $I_c(x,t)$ can be viewed as a cell density. Since the permeability $k(x,t)$, as defined in Equation S9, is now continuous there is no need to specify extra conditions at the ends of the aggregates.

To get as close as possible to modelling the aggregates as discrete entities, we want to use as large a value of a as possible. However, the larger the value of a, the finer our computational mesh needs to be to accurately capture the sharp transitions in the indicator function at the aggregate ends. If $a = 100$, then the mesh spacing in the x-direction needs to be $\Delta x < 10^{-2}$ whereas if $a = 1000$, then $\Delta x < 10^{-3}$ is required. Refining the mesh increases the size of the linear system of equations that must be solved to find the solutions of the fluid and oxygen

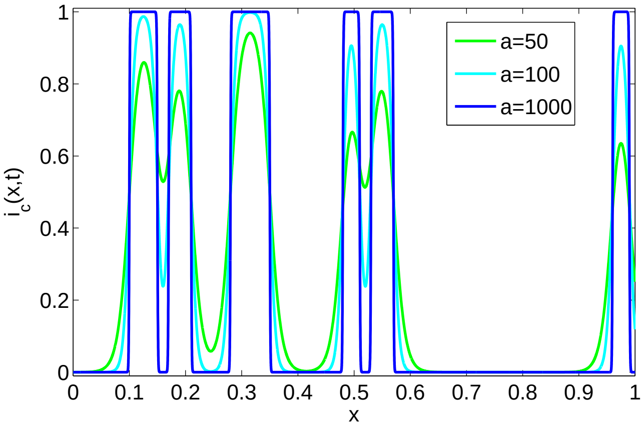

Figure S1. Smoothed cell indicator function for an example aggregate distribution. The sharpness of the transition in and out of the aggregates is shown for different values of the transition factor a.

transport problems and so increases the computation time. In practice, therefore, there is a limit on how large we can take a to be before the time taken to run simulations becomes prohibitive. For this reason we use $a = 500$ in all our simulations.

This constraint on the size of a imposes a lower limit on the aggregate length that can be used in the model, since an aggregate cannot be shorter than can be resolved with $a = 500$. This means that the minimum dimensional aggregate length that we can use is $L_{agg} = 1$mm (equivalent to roughly 25 cells). In our simulations the shortest aggregates we use are 2 mm long. We note that there is an analytical lower bound on the aggregate length, $L_{agg} > H$, necessary for the leading order growth equation (Equation (S24)) to hold, but this is a much weaker constraint than that due to the upper bound on a.

C.3 Numerical solution of the leading order system

Code was developed to solve the leading order system for the flow, oxygen transport and growth using the widely available scientific computing environment MATLAB [1].

At each time step in the code, the steady flow and oxygen transport equations ((S31)–(S32)

[1]For details see http://www.mathworks.co.uk

and (S18)–(S23) respectively) are solved for the current aggregate distribution (starting from a chosen initial distribution of N cell aggregates), and the solutions used to update the aggregate lengths via (S24). The aggregate distribution affects the flow through the dimensionless outer surface membrane permeability, $k(x,t)$, in (S31) and the oxygen transport through the nonlinear uptake term in the diffusive flux condition at the outer surface of the membrane (S20). The oxygen advection-diffusion equations are solved using the Galerkin finite element method with piecewise linear basis functions on a regular triangular mesh with 320,000 elements (160,801 nodes) (the nodes of the mesh have a dimensionless spacing of $\Delta x = 2.5 \times 10^{-3}$ in the x-direction and $\Delta y = 1.25 \times 10^{-2}$ in the y-direction, corresponding to 2.5×10^{-4} m and 2.5×10^{-6} m dimensional x- and y-spacings). To solve equations (S18)–(S23) as a linear system in this way, it is necessary to use the concentration on the upper surface of the membrane from the previous time step in the denominator of the nonlinear uptake term at each time step, i.e. to evaluate (S20) at time $t = t_n = n\Delta t$ as

$$\frac{\partial c_e^{n+1}}{\partial y} - \frac{D_m}{D_e}\frac{\partial c_m^{n+1}}{\partial y} = \gamma I_c(x,t_n)\frac{c_e^{n+1}}{c_{1/2} + c_e^n} \quad \text{on } y = h_m, \tag{S34}$$

where the superscript n denotes evaluation at time t_n. At the first time step this requires an estimate for $c^0|_{y=h_m}$. This is found by solving the oxygen transport equations (S18)–(S19), (S21)–(S23) and (S34) iteratively with the initial aggregate distribution fixed and an initial guess of $c_e|_{y=h_m} \equiv 1$ until a fixed point is reached (in practice, until the maximum difference between consecutive concentration solutions is less than 10^{-4}).

The algorithm for simulating the aggregate growth then proceeds as follows.

1. Evaluate I_c for the current aggregate distribution, $x_i(t)$ $(i = 1, \ldots, 2N)$, (the prescribed initial distribution is used at the first time step) using (S33).

2. Solve the boundary value problem for p_l given by (S31), (S9) (with I_c given by (S33)) and (S32) using the MATLAB solver `bvp4c` and evaluate the fluid velocities (S27)-(S29).

3. Solve the oxygen transport problems given by (S18) (with (S27)-(S29)) and (S19)-(S23)

using the Galerkin finite element method.

4. Calculate the growth rate of each aggregate by evaluating the right-hand side of the growth law (S24) and update the positions of the aggregate ends using an explicit Euler discretisation of the growth law. If the growth of an aggregate is larger than the space to the next aggregate either to the left or right, take the aggregate to grow until it touches the next aggregate in that direction.

5. Repeat Steps 1–4 until either the aggregates cover the entire membrane or the simulation time has elapsed.

References

1. Tharakan JP, Chau PC (1986) A radial flow hollow fiber bioreactor for the large-scale culture of mammalian cells. Biotechnology and Bioengineering 28: 329–342.

2. Korin N, Bransky A, Dinnar U, Levenberg S (2007) A parametric study of human fibroblasts culture in a microchannel bioreactor. Lab on a Chip 7: 611–617.

3. Ellis MJ, Chaudhuri JB (2007) Poly (lactic-co-glycolic acid) hollow fibre membranes for use as a tissue engineering scaffold. Biotechnology and Bioengineering 96: 177–187.

4. Yeatts AB, Fisher JP (2011) Bone tissue engineering bioreactors: dynamic culture and the influence of shear stress. Bone 48: 171–181.

5. McCoy RJ, O'Brien FJ (2010) Influence of shear stress in perfusion bioreactor cultures for the development of three-dimensional bone tissue constructs: a review. Tissue Engineering Part B: Reviews 16: 587–601.
